# Corticosteroids impair epithelial regeneration in immune-mediated intestinal damage

**DOI:** 10.1172/JCI155880

**Published:** 2024-02-13

**Authors:** Viktor Arnhold, Winston Y. Chang, Suze A. Jansen, Govindarajan Thangavelu, Marco Calafiore, Paola Vinci, Ya-Yuan Fu, Takahiro Ito, Shuichiro Takashima, Anastasiya Egorova, Jason Kuttiyara, Adam Perlstein, Marliek van Hoesel, Chen Liu, Bruce R. Blazar, Caroline A. Lindemans, Alan M. Hanash

**Affiliations:** 1 Department of Medicine, Memorial Sloan Kettering Cancer Center, New York, New York, USA.; 2 Immunology and Microbial Pathogenesis Graduate Program, Weill Cornell Medical College, New York, New York, USA.; 3 Division of Pediatrics, Regenerative Medicine Center, University Medical Center (UMC) Utrecht, Utrecht University, Utrecht, Netherlands.; 4 Department of Stem Cell Transplantation, Princess Maximá Center for Pediatric Oncology, Utrecht, Netherlands.; 5 Department of Pediatrics, Division of Blood and Marrow Transplant and Cellular Therapy, University of Minnesota, Minneapolis, Minnesota, USA.; 6 Department of Hematology, NHO Kyushu Medical Center, Fukuoka, Fukuoka, Japan.; 7 Department of Pathology, Yale School of Medicine, New Haven, Connecticut, USA.; 8 Human Oncology and Pathogenesis Program, Memorial Sloan Kettering Cancer Center, and Department of Medicine, Weill Cornell Medical College, New York, New York, USA.

**Keywords:** Immunology, Transplantation, Bone marrow transplantation, Mouse models, Mouse stem cells

## Abstract

Corticosteroid treatment (CST) failure is associated with poor outcomes for patients with gastrointestinal (GI) graft-versus-host disease (GVHD). CST is intended to target the immune system, but the glucocorticoid receptor (GR) is widely expressed, including within the intestines, where its effects are poorly understood. Here, we report that corticosteroids (CS) directly targeted intestinal epithelium, potentially worsening immune-mediated GI damage. CS administered to mice in vivo and intestinal organoid cultures ex vivo reduced epithelial proliferation. Following irradiation, immediate CST mitigated GI damage but delayed treatment attenuated regeneration and exacerbated damage. In a murine steroid-refractory (SR) GVHD model, CST impaired epithelial regeneration, worsened crypt loss, and reduced intestinal stem cell (ISC) frequencies. CST also exacerbated immune-mediated damage in organoid cultures with SR, GR-deficient T cells or IFN-γ. These findings correlated with CS-dependent changes in apoptosis-related gene expression and STAT3-related epithelial proliferation. Conversely, IL-22 administration enhanced STAT3 activity and overcame CS-mediated attenuation of regeneration, reducing crypt loss and promoting ISC expansion in steroid-treated mice with GVHD. Therefore, CST has the potential to exacerbate GI damage if it fails to control the damage-inducing immune response, but this risk may be countered by strategies augmenting epithelial regeneration, thus providing a rationale for clinical approaches combining such tissue-targeted therapies with immunosuppression.

## Abstract 2

Corticosteroids impair epithelial regeneration and can worsen immune-mediated intestinal damage but can be overcome by tissue-targeted therapies that activate STAT3.

## Graphical abstract

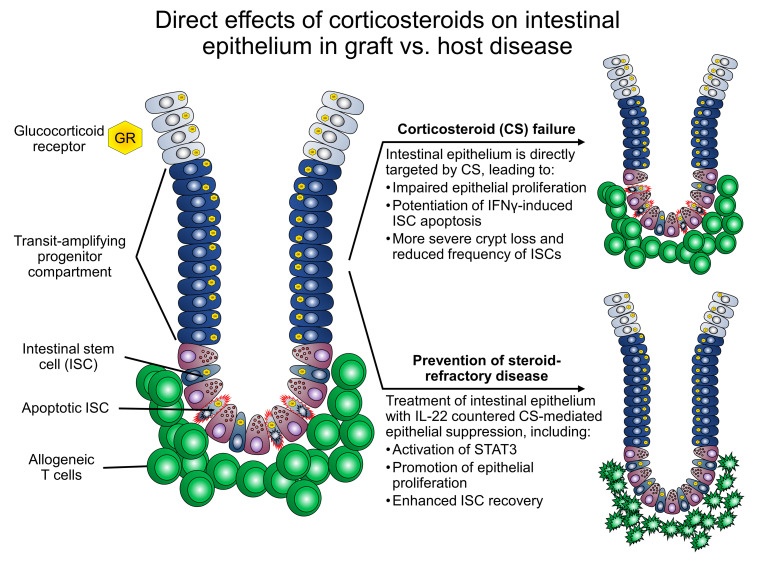

## Introduction

The epithelial lining of the gastrointestinal (GI) tract undergoes turnover every 5–7 days (1). This renewal is maintained by the cycling of leucine-rich, repeat-containing GPCR 5–positive (Lgr5^+^) intestinal stem cells (ISCs) that reside at the base of intestinal crypts (2). Cycling ISCs produce highly proliferative progenitors that move up the crypt as they divide and subsequently differentiate into the mature enterocytes of the surface epithelium. The highly proliferative cells in transition between ISCs and mature cells encompass the transit-amplifying (TA) progenitor compartment. Despite the importance of epithelial proliferation for maintenance of the intestinal lining, there is limited understanding of how this function is affected by immunosuppressive therapies administered as treatment for immune-mediated intestinal injury.

Damage to the GI tract is a frequent occurrence after allogeneic hematopoietic/bone marrow transplantation (allo-BMT). Transplant-conditioning regimens consisting of chemotherapy and/or ionizing irradiation can cause severe intestinal injury (3, 4). Furthermore, injury to the intestinal crypt epithelium is a common histopathologic finding of graft-versus-host disease (GVHD) in BMT recipients (5, 6). Acute GVHD, which occurs in 30%–70% of patients undergoing allo-BMT, is an immune-mediated complication arising from donor T cell–mediated responses against recipient tissues (7). Lower GI acute GVHD involves ileal and/or colonic mucosa, and it is associated with high morbidity and mortality (8, 9). Administration of synthetic corticosteroids (CS) is the first-line therapy for acute GVHD, with response rates ranging from 50%–70% (10). However, the optimal CS dosing for an individual patient is not always clear, and high-dose CS can be associated with substantial side effects (11–13). Additionally, CS treatment courses require tapering, leading to prolonged systemic CS exposure over several weeks. Even if CS treatment fails, CS are often continued along with other second-line agents. It is thus clinically necessary to understand broadly the direct and indirect effects of CS and their interactions with other GVHD therapies.

The therapeutic effects of CS in inflammatory settings have primarily been ascribed to their pleiotropic suppression of immune function (14). However, the GI epithelium also expresses the glucocorticoid receptor (GR) (15, 16). Little is known about the direct effects of systemic CS treatment on intestinal mucosa during GVHD-associated injury and regeneration. Utilizing in vivo murine and ex vivo murine and human organoid models, we report that CS treatment suppressed intestinal epithelial proliferation and impaired regeneration after damage, potentially exacerbating immune-mediated intestinal injury. In addition, we found that administration of exogenous IL-22 could counteract this CS-mediated toxicity, restoring epithelial regeneration and promoting intestinal recovery.

## Results

### CS reduce epithelial proliferation in vivo.

**Figure 1:**
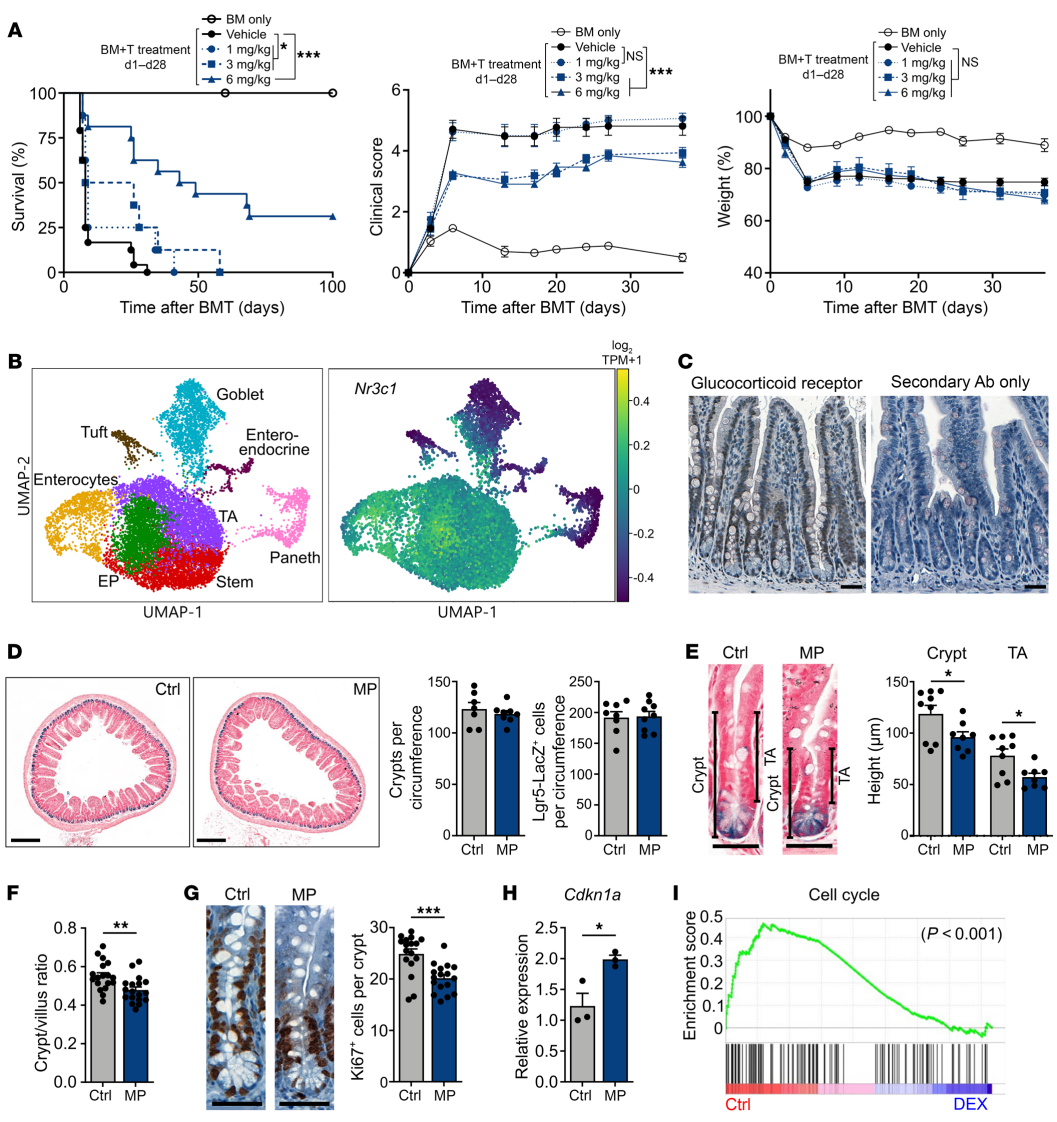
CS treatment reduces epithelial proliferation in vivo. (**A**) Survival percentage, GHVD clinical score, and relative weights of B6-into-BALB/c BMT recipients, with or without prednisolone (1, 3, or 6 mg/kg i.p. daily from day 1 to day 28 after BMT); *n* = 13 (BM only), *n* = 24 (BM plus T cells, vehicle), *n* = 8 (BM plus T cells, 1 mg/kg), *n* = 8 (BM plus T cells, 3 mg/kg), and *n* = 16 (BM plus T cells, 6 mg/kg) mice per group. Data were combined from 2 independent experiments. (**B**) UMAP visualization of 12,457 SI epithelial cells from naive WT B6 mice. Left map shows unsupervised clustering based on the expression of known marker genes; right map shows expression of *Nr3c1*. TPM, transcripts per million. (**C**) IHC images of GR staining in ileal sections from naive WT mice. Scale bars: 50 μm. (**D**–**H**) WT B6 mice were treated with MP (2 mg/kg i.p. daily for 7 days) or vehicle control (Ctrl). Results are representative of 2 experiments. (**D**) Representative *Lgr5-Lacz* images and ileal crypt and ISC frequencies (*n* = 7–9 independent sections per group). Scale bars: 250 μm. (**E** and **F**) Representative *Lgr5-Lacz* SI crypt images and data on ileal crypt height, TA height, and crypt/villus height ratio (*n* = 8–18 independent sections per group). Scale bars: 50 μm. (**G**) Ki67 IHC images and Ki67^+^ cell frequencies (*n* = 17 independent sections per group). Scale bars: 50 μm. (**H**) RT-qPCR to determine *Cdkn1a* expression in ileal tissue (*n* = 3 mice per group). (**I**) GSEA of Kyoto Encyclopedia of Genes and Genomes (KEGG) cell-cycle pathway genes in SI epithelial cells from WT mice treated with DEX or vehicle. The nominal *P* value is shown. *P < 0.05, **P < 0.01, and ***P < 0.001, by log-rank test (**A**) or 2-tailed *t* test (**D**–**H**).

In murine GVHD models, the efficacy of CS is reported to be timing and dose dependent (17, 18). Using a MHC-mismatched GVHD model, we tested early CS treatment from day 1 to day 28 after BMT at different concentrations (1, 3, or 6 mg/kg prednisolone daily). We found that CS treatment significantly improved survival in a dose-dependent manner, with high-dose CS (6 mg/kg prednisolone) increasing the median survival to 46 days, compared with 8 days in the vehicle-treated control group (Figure 1A). Lower doses of CS had less effect on median survival (Figure 1A). CS treatment also resulted in dose-dependent improvement in clinical signs of GVHD, with daily prednisolone doses of 3–6 mg/kg improving clinical GVHD scores (Figure 1A). Notably, weight loss, which may be attributed to injury of the GI epithelium during GVHD (9), was unchanged by any dose of CS treatment tested (Figure 1A). Given this discrepancy of improved survival and scoring despite persistent weight loss, we sought to scrutinize the effects of CS treatment on the intestinal epithelium.

The GR is known to be expressed across many tissues including the GI tract (14–16). To examine the expression of *Nr3c1*, the gene encoding the GR, in distinct small intestine (SI) epithelial cell populations, we analyzed a published single-cell RNA-Seq (scRNA-Seq) data set (Gene Expression Omnibus [GEO] GSE92332) of SI epithelial cells from naive WT C57BL/6 (B6) mice (19). Unsupervised clustering partitioned the cells into 8 groups associated with distinct cell types or states (Figure 1B). We found that *Nr3c1* was predominantly expressed in stem and TA cells as well as in cells of the enterocyte lineage (Figure 1B). IHC staining for the GR in the SI of WT mice confirmed GR expression in epithelial cells along the crypt/villus axis (Figure 1C).

We next investigated the effect of systemic CS administration on epithelial cells in WT mice during homeostasis. Intraperitoneal administration of methylprednisolone (MP) showed no differences in the frequencies of ileal crypts or Lgr5^+^ ISCs (20) (Figure 1D). However, we observed a significant reduction in the height of ileal crypts and the TA compartment, leading to a decrease in the crypt/villus height ratio in MP-treated animals (Figure 1, E and F). This reduction in the height of the TA compartment and the crypt/villus height ratio likely indicated a shift toward reduced proliferation within ileal epithelium. Indeed, IHC staining for Ki67 indicated a decreased frequency of proliferating cells in ileal crypts upon MP treatment (Figure 1G). MP administration also induced ileal expression of *Cdkn1a*, which encodes the cell-cycle checkpoint molecule p21 (Figure 1H). To further investigate this apparent suppression of proliferation, we performed a gene set enrichment analysis (GSEA) of SI epithelial cells from a published RNA-Seq data set (GEO GSE113691) of WT mice treated with the CS dexamethasone (DEX) or vehicle (21). Our analysis found that treatment with DEX markedly reduced proliferative cell-cycle gene expression in SI epithelium (Figure 1I). Taken together, these results indicated that systemic administration of CS did not result in obvious signs of intestinal toxicity in healthy mice at the doses tested, but it did reduce epithelial proliferation within the intestinal mucosa.

### CS exposure reduces mouse and human organoid cell proliferation.

**Figure 2:**
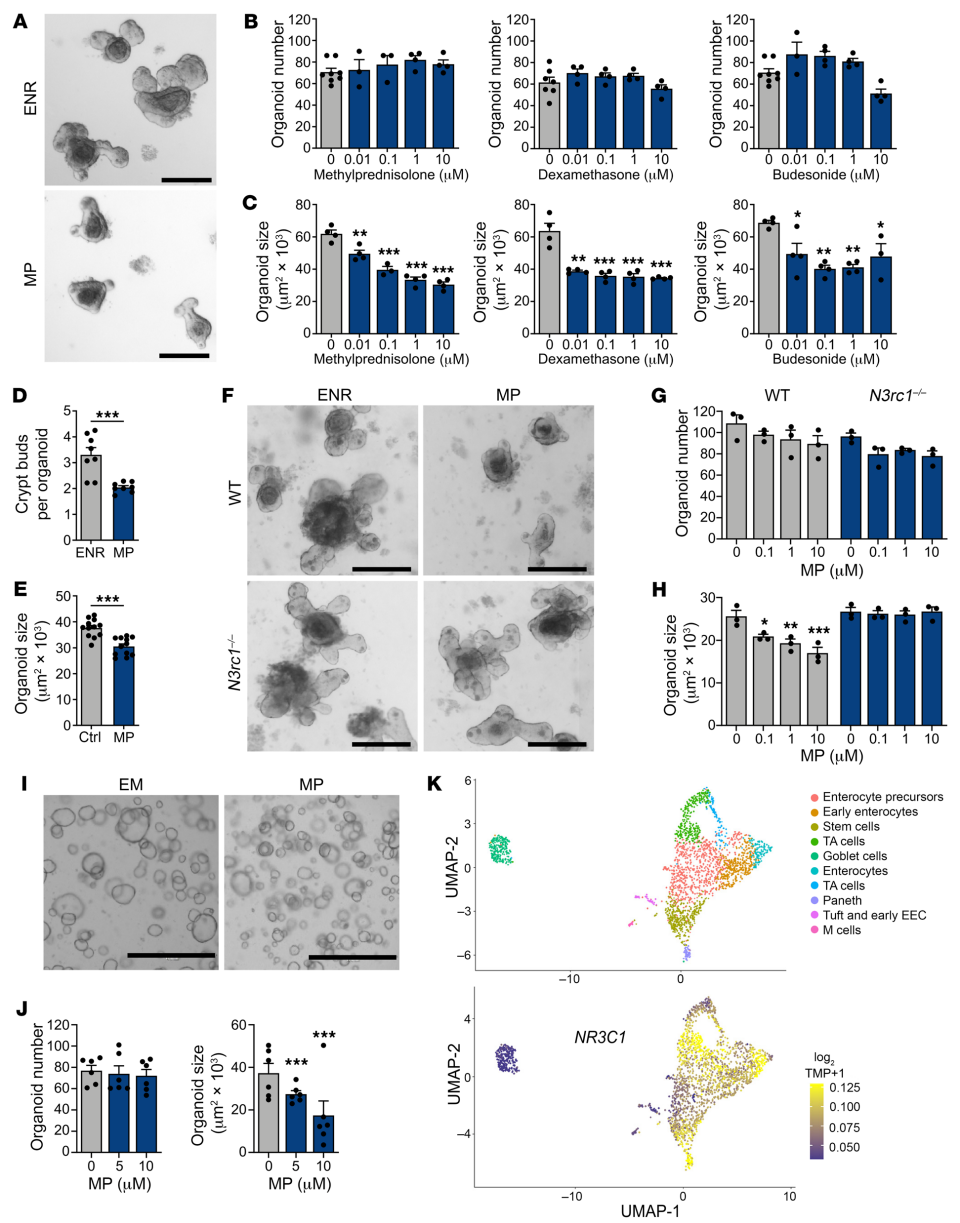
CS exposure limits the growth of murine and human SI organoids. (**A**–**C**) Representative images and frequency and size of murine SI organoids cultured in ENR with or without MP, DEX, or budesonide for 7 days (*n* = 3–8 wells per group). Scale bars: 200 μm. (**D**) Frequency of crypt bud formation in SI organoids cultured with or without MP for 5 days (*n* = 8 wells per group). (**E**) Size of SI organoids derived from harvested crypts of WT mice treated i.p. with MP or vehicle in vivo. Organoids were cultured in ENR for 6 days (*n* = 12 wells per group). (**F**–**H**) Representative images and frequency and size of WT and *Nr3c1^–/–^* SI organoids cultured with or without MP for 4 days (*n* = 3 wells per group). Scale bars: 200 μm. (**I** and **J**) Representative images and frequency and size of human SI organoids cultured with or without MP (*n* = 6 wells per group). Scale bars: 1,000 μm. (**K**) UMAP visualization of 2,342 human epithelial cells from ileal crypts. Top map shows unsupervised clustering based on the expression of known marker genes. Bottom map shows the expression of *NR3C1*. EEC, enteroendocrine cells. *P < 0.05, **P < 0.01, and ***P < 0.001, by 2-tailed *t* test or 1-way ANOVA. Data are representative of at least 2 independent experiments or were combined from 2 independent experiments (**I** and **J**).

Interpreting the effects of systemically administered CS can be challenging due to the numerous potential CS targets in vivo, including epithelial, stromal, and hematopoietic cells. To address this limitation, we used murine ex vivo crypt–derived SI organoid cultures to explore the direct effects of CS on intestinal epithelium. After exposure to CS, organoids were evaluated for size and frequency, which assess epithelial proliferation and viability, respectively (22). Testing of several clinically relevant CS agents demonstrated that the addition of MP, DEX, or budesonide to standard EGF/noggin/R-spondin 1 (ENR) culture conditions reduced murine organoid size without affecting organoid numbers (Figure 2, A–C). CS treatment also attenuated organoid crypt bud formation (Figure 2D). MP pretreatment in vivo prior to crypt isolation caused a significant reduction in organoid size as well in comparison with cultures derived from untreated mice (Figure 2E). We also found that *Nr3c1^–/–^* organoids were resistant to growth inhibition by MP, further indicating that CS could directly act on intestinal epithelial cells through the GR to suppress growth (Figure 2, F–H).

We next examined the effects of CS on human intestinal tissue. Using ex vivo organoid cultures generated from primary duodenal tissue, we found that MP decreased the size of human intestinal organoids without affecting organoid frequency (Figure 2, I and J). Furthermore, we examined *NR3C1* expression in human SI epithelial cell populations by analyzing a published scRNA-Seq data set (GEO GSE119969, GSM3389578) of EpCAM^+^ epithelial cells from human SI tissue (23). Consistent with our scRNA-Seq analysis of murine SI epithelial cells (Figure 1B), *NR3C1* expression was detected in stem, TA, and enterocyte lineage cells (Figure 2K). Overall, studies using both mouse and human models indicated that CS directly suppressed intestinal organoid growth, and this appeared to occur in a GR-dependent manner.

**Figure 3:**
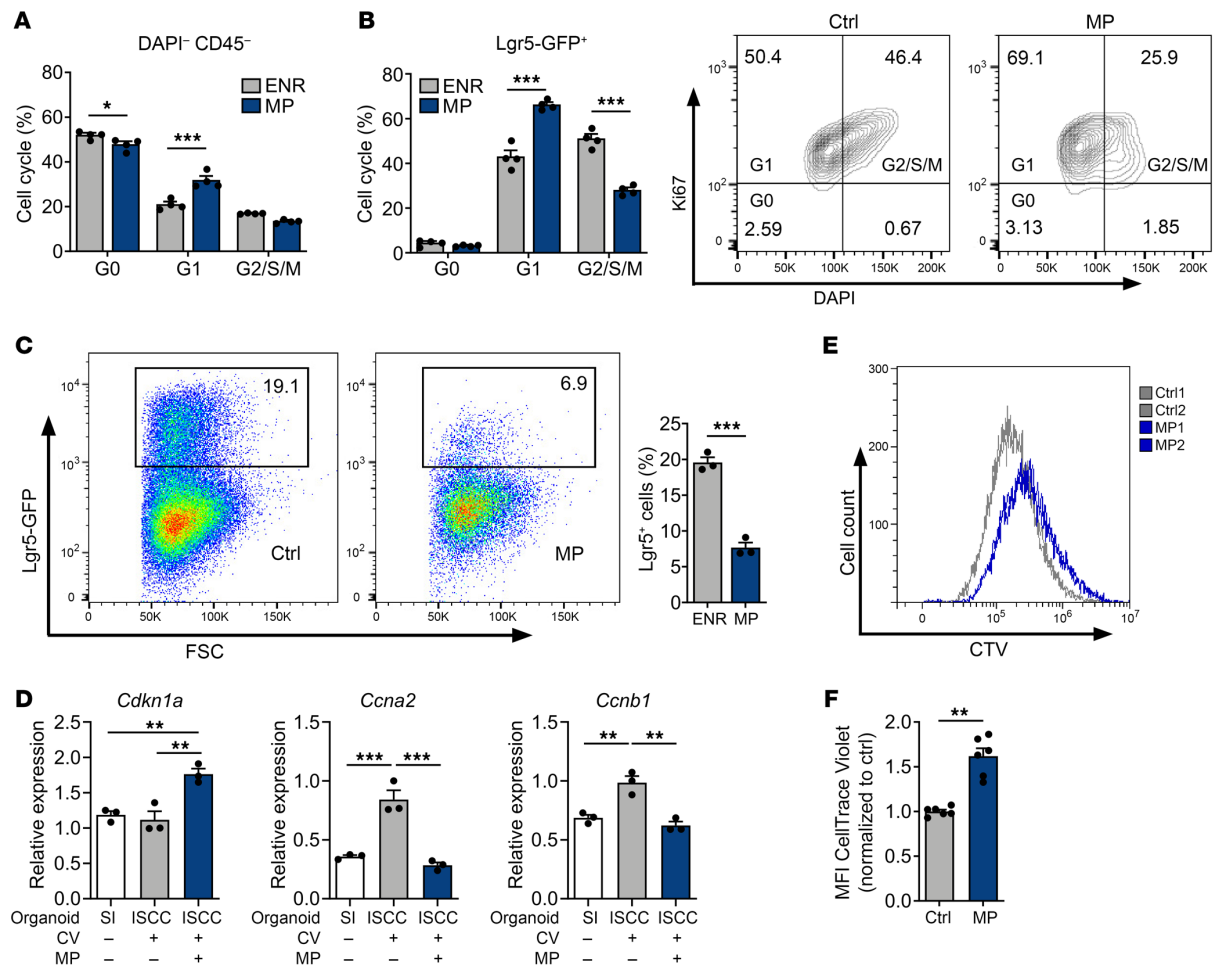
CS reduce the proliferation of murine and human organoid cells. (**A**) Quantifications of intracellular Ki67-DAPI cell-cycle analysis in live organoid cells cultured with or without MP (10 μM) for 5 days (*n* = 4 wells per group). (**B**) Flow cytometry plots and quantification of intracellular Ki67-DAPI cell-cycle analysis in GFP^+^ cells from *Lgr5-GFP* SI organoids cultured with or without MP (10 μM) for 5 days (*n* = 4 wells per group). (**C**) Flow cytometry plots and quantification of GFP^+^ cell fractions from *Lgr5-GFP* SI organoids cultured with or without MP (10 μM) for 5 days (*n* = 3 wells per group). (**D**) RT-qPCR to determine *Cdkn1a*, *Ccna2*, and *Ccnb1* expression in organoids derived from SI crypts cultured in ENR or ISC colonies (ISCC) cultured in ENR supplemented with histone deacetylase and GSK3β inhibition (CV) with or without MP (10 μM) for 4 days (*n* = 3 wells per group). (**E** and **F**) Flow cytometry plots and quantification of CTV in human SI organoids cultured with or without MP (10 μM) for 5 days (*n* = 6 donors per group). *P < 0.05, **P < 0.01, and ***P < 0.001, by 2-tailed *t* test or 1-way ANOVA. Data are representative of at least 2 independent experiments.

In order to evaluate the effects of CS on epithelial proliferation more precisely, we performed flow cytometric Ki67-DAPI cell-cycle analysis using steroid-treated murine organoids. MP treatment had a substantial effect on cell cycling, increasing the proportion of cells in the G_1_ phase (Figure 3A), potentially reflecting the presence of steroid-induced G_1_ arrest. To examine ISCs specifically, we also evaluated cell-cycle distributions in organoids derived from *Lgr5-GFP-IRES-CreERT2* (*Lgr5-GFP*) mice. ISCs are a constitutively active stem cell population (20), and few were found to be in G_0_ (Figure 3B). Following MP treatment, the proportion of Lgr5-GFP^+^ ISCs in G_1_ increased, and the proportion in G_2_/S/M decreased (Figure 3B), suggesting reduced stem cell progression through the cell cycle in the presence of CS. Consistent with this, we also found that MP treatment reduced ISC frequency within organoids (Figure 3C), likely due to reduced ISC proliferation.

To further evaluate the direct effects of CS on ISC proliferation, we generated highly enriched “ISC colonies” by sorting ISCs from *Lgr5-GFP* mice and culturing them in the presence of glycogen synthase kinase 3β (GSK3β) and histone deacetylase (HDAC) inhibitors, which enhances Wnt and Notch signaling and drives stem cell expansion (24). Exposure of these ISC colonies to MP increased the expression of *Cdkn1a* and reduced the expression of the cyclin genes *Ccna2* and *Ccnb1* (Figure 3D), indicating antiproliferative transcriptional changes in the ISCs. Together, the cell-cycle changes, transcriptional changes, and reduced ISC frequency after MP treatment ex vivo indicated that CS could suppress murine ISC proliferation. Additionally, in human organoids examined with the CellTrace Violet (CTV) proliferation assay, MP treatment led to greater retention of the fluorescent dye compared with untreated organoids. This provided further evidence that CS directly reduced epithelial proliferation, including within human intestinal epithelium (Figure 3, E and F).

### Epithelial effects of CS after radiation injury are timing dependent.

**Figure 4:**
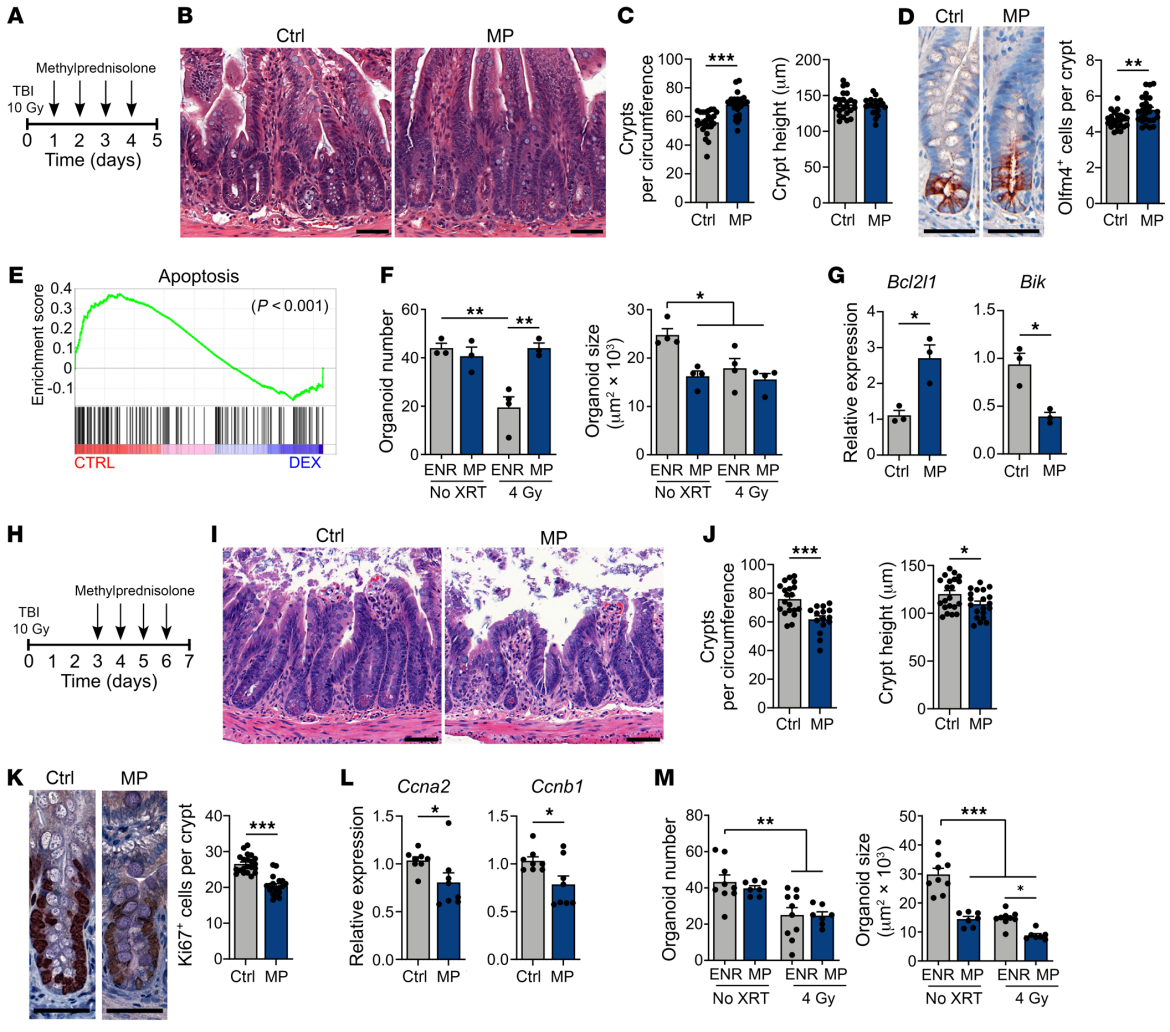
Epithelial effects of CS treatment after irradiation are timing dependent. (**A**–**D**) WT B6 mice were treated with MP (2 mg/kg) or vehicle i.p. daily starting 24 hours after TBI. (**B** and **C**) Representative images, ileal crypt frequency, and height, 5 days after TBI (*n* = 21–25 sections per group). Scale bars: 50 μm. (**D**) Representative Olfm4 IHC staining and Olfm4^+^ cell frequencies 5 days after TBI (*n* = 25–27 sections per group). Scale bars: 50 μm. (**E**) GSEA of the MSigDB apoptosis gene set in SI epithelial cells from WT mice treated with DEX or vehicle. One analysis and a nominal *P* value are shown. (**F** and **G**) SI crypt cells were plated 4 hours prior to 4 Gy irradiation. Cultures were treated with MP (10 μM) 16 hours after irradiation. (**F**) Organoids were evaluated for frequency and size 3 days after irradiation (*n* = 3–4 wells per group). (**G**) *Bcl2l1* and *Bik* expression was determined by RT-qPCR 48 hours after irradiation (*n* = 3 wells per group). (**H**–**L**) WT B6 animals were treated with MP (2 mg/kg) or vehicle i.p. daily, starting 72 hours after TBI. (**I** and **J**) Representative images and ileal crypt frequency and height (*n* = 15–21 sections per group), 7 days after TBI. Scale bars: 50 μm. (**K**) Representative Ki67 IHC images and data showing Ki67^+^ cell frequencies, 7 days after TBI (*n* = 19–21 sections per group). Scale bars: 50 μm. (**L**) RT-qPCR showing *Ccna2* and *Ccnb1* expression in enriched SI crypts, 7 days after TBI (*n* = 8 animals per group). (**M**) SI crypt cells were plated 4 hours prior to 4 Gy irradiation; cultures were treated with MP (10 μM) 3 days after irradiation. Seven days after irradiation, organoids were evaluated for frequency and size (*n* = 7–10 wells per group). *P < 0.05, **P < 0.01, and ***P < 0.001, by 2-tailed *t* test or 1-way ANOVA. Data are representative of at least 2 independent experiments or were combined from 2 independent experiments (**A**–**D** and **H**–**L**).

We next investigated the consequences of CS administration for radiation-induced intestinal injury and regeneration. Following radiation injury, surviving crypts become highly proliferative. This proliferation can typically be observed 3–5 days after total body irradiation (TBI) (25, 26). Treatment of irradiated mice with MP starting 24 hours after TBI mitigated epithelial injury 4 days later, as evidenced by increased preservation of ileal crypt frequencies (Figure 4, A–C). In this tissue-protective setting, there was no difference in crypt height detected in response to MP treatment by day 5 after TBI (Figure 4C). Staining for the SI ISC marker olfactomedin 4 (Olfm4) (27) indicated that MP treatment also protected the stem cells from radiation injury (Figure 4D). To investigate how CS may mitigate radiation injury, we again examined the RNA-Seq GSEA of intestinal epithelial cells from naive mice treated with DEX (GEO GSE113691) (21). DEX treatment was associated with reduced expression of a proapoptotic gene signature (the Molecular Signatures Database [MSigDB] apoptosis gene set) compared with control epithelium (Figure 4E), suggesting a potential mechanism by which CS treatment could reduce intestinal radiation injury. To further examine this potential to protect epithelium from radiation injury, we isolated SI crypts for organoid culturing, irradiated them, and then treated them with CS. Similar to our in vivo findings, MP treatment within 24 hours after irradiation preserved organoid frequencies and had no discernible effect on their size (Figure 4F). This MP treatment also increased expression of the antiapoptotic gene *Bcl2l1* and decreased expression of the proapoptotic gene *Bik* within SI organoids after irradiation (Figure 4G).

In contrast to early steroid administration, delaying the MP treatment until days 3–6 after TBI (Figure 4H), during the proliferative phase of the epithelial response to radiation injury, led to increased tissue pathology with worsened ileal crypt loss (Figure 4, I and J). MP treatment starting on day 3 after TBI also reduced crypt heights and Ki67^+^ cell frequency, indicating attenuated epithelial proliferation (Figure 4, J and K), and ileal expression of *Ccna2* and *Ccnb1* was reduced as well in MP-treated animals (Figure 4L). Similarly, in the organoid model, delaying the initiation of MP treatment until day 3 after crypt irradiation reduced organoid sizes and did not preserve organoid frequencies (Figure 4M). Overall, our in vivo and ex vivo studies indicated that early initiation of MP treatment could mitigate the severity of intestinal radiation injury, but delayed treatment failed to do so and instead impaired the regenerative response.

### CS impair the epithelial response to immune-mediated GI damage.

**Figure 5:**
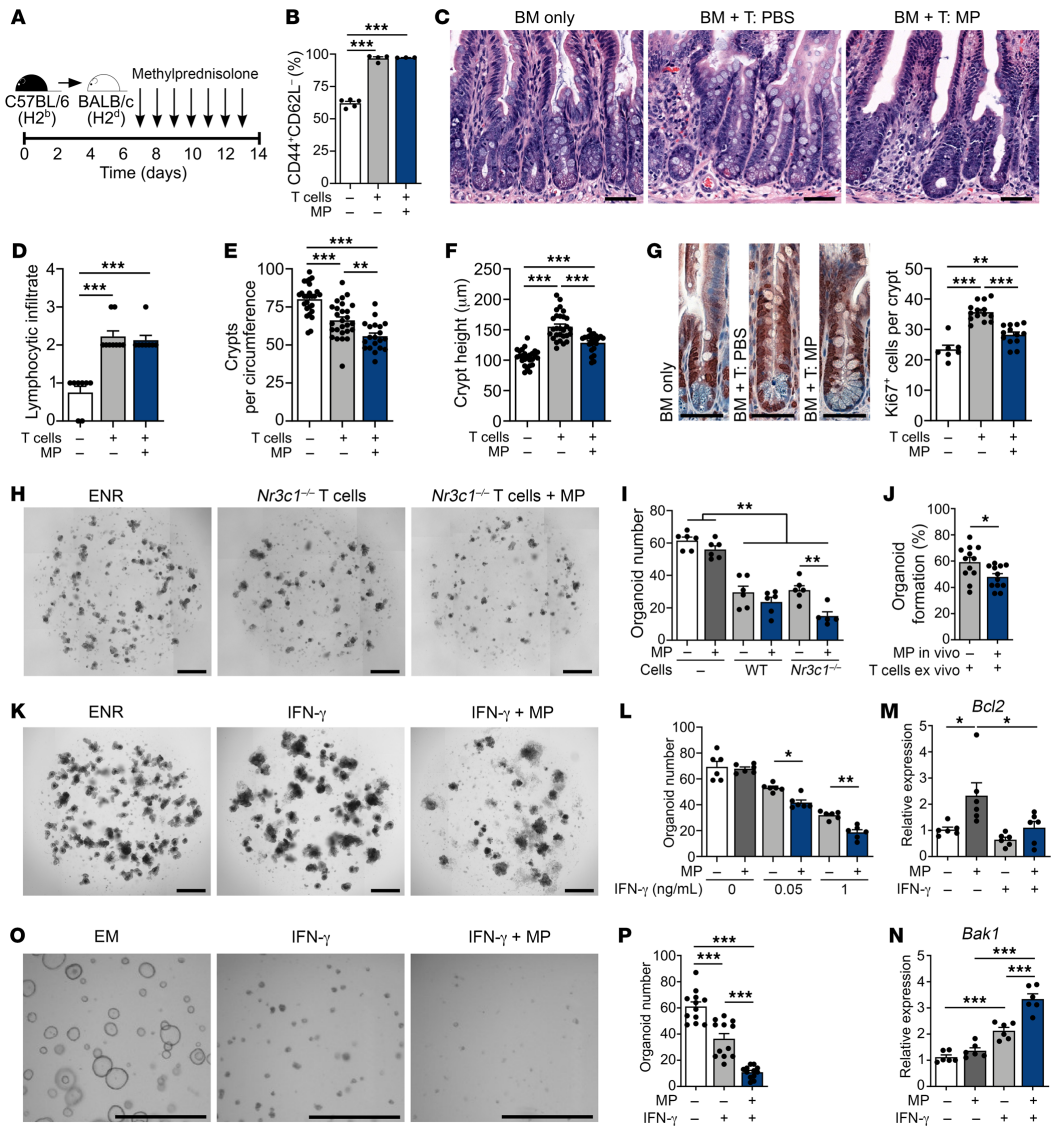
CS impair epithelial regeneration, increasing the severity of in vivo and ex vivo immune-mediated GI damage. (**A**–**G**) B6-into-BALB/c transplantation of BM with or without T cells; recipient mice were treated with MP (2 mg/kg) or vehicle i.p. daily, starting on day 7 through day 14 after BMT. Recipient mice were evaluated on day 14 after BMT. (**B**) CD44^+^CD62L^–^ cell proportion of CD45^+^CD3^+^CD4^+^CD8^–^ splenocytes (*n* = 3–5 mice per group). (**C**) Representative images of ileum. Scale bars: 50 μm. (**D**) SI lymphocytic infiltrate histopathology score (*n* = 8–9 mice per group). (**E** and **F**) Ileal crypt frequency and height (*n* = 20–28 sections per group). Scale bars: 50 μm. (**G**) Representative images and Ki67^+^ cell frequency (*n* = 7–14 sections per group). (**H** and **I**) Representative images and B6 SI organoid frequency after culturing with or without anti-CD3/CD28–activated *Nr3c1^fl/fl^* or *Nr3c1^fl/fl^* *Cd4-Cre* B6 T cells with or without MP (10 μM) for 4 days (*n* = 5–6 wells per group). Scale bars: 500 μm. (**J**) B6 SI organoid frequency after in vivo MP (or vehicle) treatment prior to crypt isolation and subsequent culturing with anti-CD3/CD28–activated WT B6 T cells on day 6 of culturing (*n* = 12 wells per group). (**K** and **L**) Representative images and organoid frequency after culturing with or without MP (10 μM) and rmIFN-γ for 6 days (*n* = 6 wells per group). Scale bars: 500 μm. (**M** and **N**) qPCR to determine *Bcl2* and *Bak1* expression in organoids cultured with or without MP (10 μM) and rmIFN-γ (1 ng/mL) for 3 days (*n* = 6 wells per group). (**O** and **P**) Representative images and human organoid frequency after culturing with or without MP (10 μM) and rhIFN-γ (2 ng/mL) for 7 days (*n* = 12 fields of view in 6 wells per group). Scale bars: 1,000 μm. *P < 0.05, **P < 0.01, and ***P < 0.001, by 2-tailed *t* test or 1-way ANOVA. Data are representative of at least 2 independent experiments or were combined from 2 experiments (**A**–**G**).

We next investigated the effects of CS on intestinal epithelium during immune-mediated injury occurring after hematopoietic transplantation. B6-into-BALB/c MHC-mismatched allo-BMT results in CD4^+^ T cell–driven GVHD (28), with donor T cell infiltration and tissue pathology established in the intestines within 4–7 days after BMT (29). To study the effects of CS on intestinal epithelium in a setting after the alloreactive injury process had begun, daily administration of MP was initiated on day 7 after BMT (Figure 5A). As evidence of active GVHD at the time of treatment initiation, recipients transplanted with allogeneic BM and T cells exhibited increased clinical GVHD scores and a mean weight loss of over 20% on day 7 after BMT (Supplemental Figure 1A; supplemental material available online with this article; https://doi.org/10.1172/JCI155880DS1). The effects of CS treatment were then assessed following a clinically modeled 7-day course of 2 mg/kg MP administered daily, as would typically be given to patients for initial treatment of acute GVHD involving the lower GI tract (Figure 5A).

After 1 week of treating the transplanted mice, their systemic GVHD scores remained elevated and their weight loss persisted, and we observed no improvement in these parameters in MP-treated BMT recipients compared with vehicle-only controls (Supplemental Figure 1B), thus indicating clinical features that would meet criteria for steroid-refractory (SR) GVHD (10). Furthermore, despite the MP treatment course, splenic T cells maintained an activated effector phenotype (Figure 5B), and lymphocyte infiltration persisted in the intestines (Figure 5, C and D). Additionally, B6-into-BALB/c allo-BMT recipients treated with a prolonged 4-week course of daily 2 mg/kg MP beginning on day 7 after transplantation continued to show no improvement in survival, scores, or weight loss (Supplemental Figure 1C). Therefore, delayed MP treatment in this GVHD model was not effective in restraining the allogeneic immune response, as systemic findings, T cell activation, and lymphocytic infiltration all remained unabated and indicated the presence of SR GVHD.

Next, examination of the intestinal mucosa from mice transplanted with allogeneic T cells identified ileal crypt loss, increased length of the residual crypts, and increased Ki67^+^ cell frequency within those crypts compared with BM-only controls (Figure 5, E–G), indicating the presence of damage-associated epithelial regeneration in BMT recipients with GVHD. While MP treatment did not affect T cell phenotype or intestinal infiltration (Figure 5, B–D), the epithelial proliferative response was attenuated, and we noted more severe intestinal GVHD pathology (Figure 5, E–G). MP-treated mice demonstrated exacerbated crypt loss (Figure 5E) in association with reduced crypt height (Figure 5F) and reduced Ki67^+^ cell frequency (Figure 5G) compared with vehicle-treated allo-BMT recipients. Therefore, in a setting where CS failed to suppress the pathophysiologic alloreactive immune response in GVHD, CS did appear to suppress intestinal epithelial regeneration and exacerbate GVHD-mediated tissue injury.

To examine the role of epithelium-specific GR signaling within the SI during GI GVHD, we generated *Nr3c1^fl/fl^* *Olfm4-CreERT2* (*Nr3c1^ΔIEC^*) mice, in which the GR was genetically deleted in SI ISCs (and their ensuing progeny) following tamoxifen treatment. As a result of GR deletion in ISCs, the deletion was propagated throughout the SI epithelium as it underwent turnover (Supplemental Figure 2A). GR deletion was well tolerated, with no effect on crypt frequency at baseline (Supplemental Figure 2B). *Nr3c1^ΔIEC^* mice were then used as transplant recipients in a B10.BR-into-B6 MHC-mismatched allo-BMT model to evaluate the effect of endogenous glucocorticoids and GR function within the epithelium during GVHD. After transplantation, we found that *Nr3c1^ΔIEC^* mice had reduced intestinal injury when compared with WT recipients, with substantial preservation of crypt frequencies (Supplemental Figure 2, C and D). Thus, in addition to the increased intestinal injury observed following exogenous MP treatment in our SR GVHD model, endogenous epithelial GR expression within the SI contributed to intestinal GVHD pathology.

### CS augment the immune-mediated GI damage induced by T cells and their effector cytokines ex vivo.

To further characterize the effects of CS on epithelial cells during immune-mediated damage, we used an ex vivo model of intestinal organoids cultured with activated T cells (30). In order to focus on the epithelial effects of CS and control for effects on T cells, coculturing was performed using either GR-intact (*Nr3c1^fl/fl^*) or GR-deficient (*Nr3c1^fl/fl^* *Cd4-Cre*) T cells (Figure 5, H and I). Addition of MP on its own again had no effect on organoid viability, while coculturing with either GR-intact or GR-deficient T cells resulted in substantial organoid loss (Figure 5I). Addition of MP to cocultures with GR-intact T cells, where both the organoids and T cells could be targeted by CS, had no discernible effect on organoid frequency (Figure 5I). In contrast, addition of MP to cocultures with GR-deficient T cells, where GR expression was restricted to the epithelium, resulted in more severe organoid reductions (Figure 5, H and I). These results suggested that MP could act directly on epithelium and increase sensitivity to T cell–mediated damage. Similarly, persistent steroid-dependent epithelial sensitivity was observed in T cell coculture experiments using SI crypts isolated from mice treated with MP or vehicle. Pretreatment with MP in vivo resulted in reduced organoid frequencies ex vivo following coculturing with T cells (Figure 5J). Therefore, ex vivo studies indicated that epithelial exposure to CS could potentiate the severity of immune-mediated GI damage.

T cell–derived IFN-γ contributes to crypt loss in GVHD and organoid toxicity ex vivo, directly inducing ISC apoptosis (30). We thus exposed intestinal organoids to MP and IFN-γ concurrently to evaluate their combined effect and potential relevance to CS-potentiated GI damage. Similar to findings with GR-deficient T cells, exposure to MP exacerbated IFN-γ–driven organoid loss (Figure 5, K and L). We next investigated apoptosis-related gene expression within organoids treated with IFN-γ and/or MP. Consistent with our earlier observations, MP treatment alone increased expression of the antiapoptotic genes *Bcl2* and *Bcl2l1* in SI organoids (Figure 5M and Supplemental Figure 3A). In contrast, gene expression following exposure to IFN-γ appeared more proapoptotic, with increased *Bak1* and trends toward reductions in *Bcl2* and *Bcl2l1* (Figure 5, M and N, and Supplemental Figure 3A). During concurrent treatment with MP and IFN-γ, the CS-associated upregulation of *Bcl2* and *Bcl2l1* expression was attenuated, while IFN-γ–associated *Bak1* upregulation was elevated further (Figure 5, M and N, and Supplemental Figure 3A). These findings thus indicated that CS exposure could yield distinct transcriptional profiles in epithelial cells on its own versus in combination with cytotoxic cytokines, potentially leading to an enhanced proapoptotic tissue response during immune-mediated injury. Consistent with these findings, concurrent exposure to MP and IFN-γ also reduced the frequency of viable human SI organoids more than exposure to IFN-γ alone (Figure 5, O and P). Therefore, experiments modeling immune-mediated GI damage in vivo and ex vivo indicated that CS could have direct effects on tissues, exacerbating the damage induced by T cells and their effector cytokines.

### IL-22 treatment overcomes CS-induced inhibition of epithelial proliferation.

Ruxolitinib, a JAK1/2 inhibitor, is FDA approved for the treatment of SR GVHD (31). Although the rationale for its use in GVHD is largely based on its potential for targeting alloreactive T cells, it has also been shown that ruxolitinib can interfere with epithelial JAK/STAT signaling (30). To test whether ruxolitinib could affect CS-mediated suppression of epithelial regeneration, we treated ex vivo organoid cultures with ruxolitinib and MP. Analysis of organoid size indicated that ruxolitinib was unable to inhibit CS-mediated suppression of organoid growth, and it may even potentiate it (Supplemental Figure 3B). Therefore, we sought to scrutinize the relationship between JAK/STAT signaling and CS in the suppression of epithelial regeneration and to examine alternative approaches for protecting epithelium from steroids.

STAT3 can contribute to epithelial regeneration and recovery from intestinal injury (22, 32), and it has been reported that the GR can interfere with STAT3 function, either directly by binding to it, or indirectly by binding to genetic regulatory elements (33). Therefore, we investigated whether CS-mediated suppression of epithelial proliferation could involve impairment of STAT3 function. First, we cultured WT organoids with MP and Stattic, a STAT3 inhibitor. While Stattic itself suppressed organoid growth, as previously described (22), addition of MP did not further decrease organoid size (Supplemental Figure 4A). Next, organoids derived from *Stat3^fl/fl^* *Villin-Cre* (*Stat3^ΔIEC^*) mice, which lack STAT3 in the intestinal epithelium, were exposed to MP. Once again, we found that MP failed to suppress epithelial growth when STAT3 was already impaired, as the size of STAT3-deficient organoids was unchanged in the presence of MP (Supplemental Figure 4B). Together, these findings suggested that CS-mediated suppression of intestinal regeneration may have been due in part to inhibition of STAT3. These findings also suggested that promotion of STAT3 function could potentially reverse some of the epithelial suppression induced by CS.

**Figure 6:**
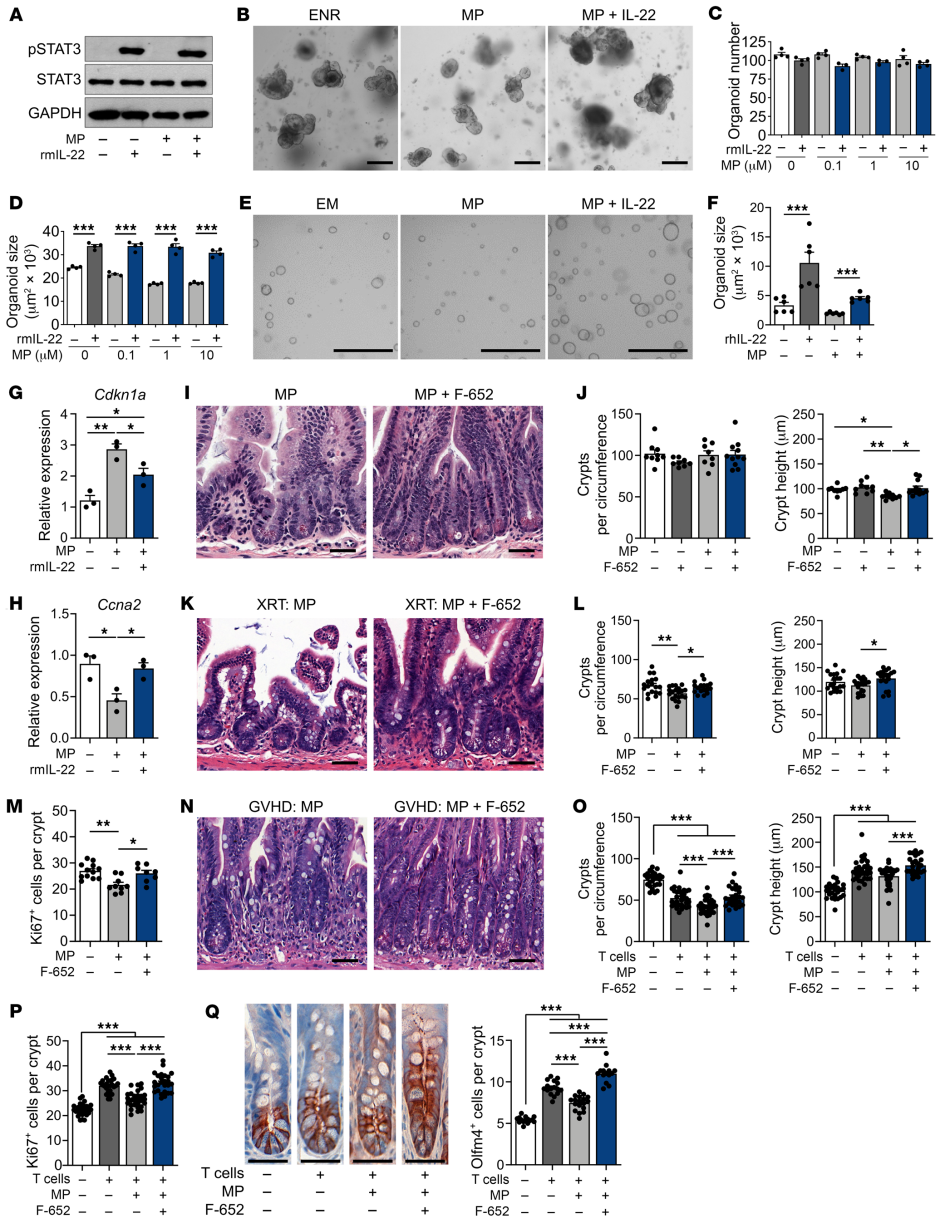
IL-22 administration overcomes CS-mediated inhibition of epithelial proliferation ex vivo and in vivo. (**A**) Representative Western blot of SI organoids treated with or without MP (10 μM) for 24 hours, followed by treatment with rmIL-22 (5 ng/mL) for 2 hours. (**B**–**D**) Representative images and frequency and size of SI organoids cultured with or without MP and rmIL-22 (0.5 ng/mL) for 5 days (*n* = 3–4 wells per group). Scale bars: 200 μm. (**E** and **F**) Representative images and size of human SI organoids cultured with or without MP (10 μM) and rhIL-22 (10 ng/mL) for 6 days (*n* = 6 wells per group). Scale bars: 1,000 μm. (**G** and **H**) qPCR to determine *Cdkn1a* and *Ccna2* expression in organoids cultured with or without MP (10 μM) and rmIL-22 (1 ng/mL) for 3 days (*n* = 3 wells per group). (**I** and **J**) B6 mice treated or not with MP (2 mg/kg i.p. daily) with or without F-652 (100 μg/kg s.c. every other day). Representative images and ileal crypt frequency and height on day 7 (*n* = 8–12 independent sections per group). Scale bars: 50 μm. (**K**–**M**) WT B6 mice were treated or not with MP (2 mg/kg i.p. daily) with or without F-652 (100 μg/kg s.c. every other day), starting 72 hours after TBI. Representative images, ileal crypt frequency and height, and Ki67^+^ cell frequency (*n* = 8–23 independent sections per group) on day 7. Scale bars: 50 μm. (**N**–**Q**) B6-into-BALB/c transplantation of BM with or without T cells. Recipients were treated or not with MP (2 mg/kg i.p. daily) with or without F-652 (100 μg/kg s.c. every other day), starting on day 7 after BMT. Ileal crypt frequency and height and Ki67^+^ and Olfm4^+^ cell frequency (*n* = 14–31 independent sections per group) on day 14 after BMT. Scale bars: 50 μm. Data are representative of at least 2 independent experiments or were combined from 2 experiments (**K**–**Q**). *P < 0.05, **P < 0.01, and ***P < 0.001, by 2-tailed *t* test or 1-way ANOVA.

IL-22 has been shown to activate STAT3 and promote epithelial recovery in experimental GVHD models (22, 34), and it has recently been investigated in a clinical trial for the treatment of newly diagnosed GI GVHD along with systemic CS (35). Here, we evaluated the effect of their combined treatment on STAT3 phosphorylation ex vivo via Western blot analysis of murine SI organoids, which indicated intact epithelial STAT3 activation by recombinant murine IL-22 (rmIL-22) in the presence of MP (Figure 6A). We thus investigated whether IL-22 treatment could promote epithelial regeneration in the presence of CS. Although cultures combining IFN-γ and MP had increased toxicity (Figure 5, K and L), organoid frequencies remained stable upon treatment with IL-22 and MP, and rmIL-22 treatment was able to augment the growth and size of murine organoids despite the presence of MP (Figure 6, B–D). Furthermore, recombinant human IL-22 (rhIL-22) promoted the growth of human SI organoids cultured in the presence of MP as well (Figure 6, E and F). Consistent with these observations, IL-22 treatment attenuated the MP-induced upregulation of *Cdkn1a* and downregulation of *Ccna2* in cocultured organoids (Figure 6, G and H).

Next, we evaluated the potential of F-652, a clinical-grade rhIL-22 dimer/Fc-fusion protein, to overcome MP-mediated suppression of epithelial proliferation in vivo. During homeostasis, F-652 and MP administration had no effect on ileal crypt frequencies when administered alone or in combination (Figure 6, I and J), which was consistent with the stable organoid frequencies observed with ex vivo treatment. Also consistent with the ex vivo findings, treatment in vivo with F-652 reversed the MP-mediated reduction in ileal crypt height (Figure 6J). We then tested the combination of F-652 plus MP treatments in models of GI damage. In radiation injury, daily MP administration for 4 days, starting on day 3 after TBI, once again worsened radiation-associated crypt loss and reduced regeneration-associated Ki67^+^ cell frequency (Figure 6, K–M). However, adding F-652 increased ileal crypt recovery, crypt height, and Ki67^+^ cell frequency (Figure 6, K–M), indicating an enhanced regenerative capacity of intestinal crypts despite the administration of CS.

Finally, we assessed combined CS and F-652 treatment in GVHD. As expected, B6-into-BALB/c BMT with allogeneic T cells was associated with weight loss and systemic clinical scoring indicative of GVHD (Supplemental Figure 5). Recipients in this model treated with MP, 2 mg/kg daily starting on day 7 after transplantation, trended toward increased systemic GVHD scoring by day 14 after transplantation, which was not observed in the mice that also received F-652 during the same time frame. Upon histologic assessment, coadministration of F-652 was found to prevent CS-associated crypt loss, augment crypt height, and augment Ki67^+^ cell frequency as well (Figure 6, N–P). Furthermore, assessment of Olfm4^+^ ISCs in this model indicated that, while MP treatment on its own decreased stem cell frequencies, the addition of F-652 increased them (Figure 6Q). Therefore, these findings indicate that F-652 administration could reduce the severity of intestinal injury and augment epithelial regeneration during CS treatment for GVHD.

## Discussion

In this study, we found that CS directly suppressed epithelial proliferation within the intestines. In the setting of TBI, CS treatment had antiapoptotic and antiproliferative effects on the intestinal epithelium, which correlated with protection from tissue damage or exacerbation of tissue damage depending on the timing of treatment. In models of immune-mediated intestinal injury in vivo and ex vivo intended to recapitulate features of SR GVHD, CS suppressed epithelial proliferation and exacerbated T cell– and IFN-γ–mediated epithelial damage. Ex vivo, CS-mediated suppression of epithelial proliferation appeared to involve impairment of STAT3 function. IL-22 activated STAT3 and was able to overcome the effects of CS in organoids and in vivo, improving epithelial regeneration and reversing the steroid-associated exacerbation of intestinal injury identified after TBI and BMT.

In studying CS-mediated suppression of epithelial proliferation, we found that MP suppressed ISC proliferation and reduced the stem cell frequency within intestinal organoids. This reduction in ISC frequencies was not apparent when normal/healthy mice were treated with MP, but crypt heights and crypt/villus height ratios in these mice were reduced, indicating that the overall precursor compartment was indeed diminished. This diminishment in the overall precursor pool thus included a relative reduction in the proportion of epithelial precursors (crypt-resident) to mature enterocytes (villus-resident), which is somewhat similar to the relative reduction of ISC frequencies as a percentage of organoid cells following ex vivo MP treatment. Furthermore, in vivo reductions in ISC frequencies within crypts became evident after damage in GVHD mice treated with MP. Our experiments implicated direct CS regulation of the epithelium through the GR as a driver of altered ISC cycling and reduced epithelial proliferation, although it is possible that indirect mechanisms mediated through immune effectors or the ISC niche could have contributed in vivo as well.

Scrutinizing the effects of CS on epithelial injury and regeneration after TBI uncovered opposing results depending on the timing of treatment. While early treatment (the day after TBI) mitigated radiation-induced SI epithelial injury, delaying treatment by 2 days (until day 3 after TBI) led to suppressed crypt regeneration and impaired epithelial recovery. These findings were recapitulated in ex vivo experiments, in which MP was added to organoid cultures at different time points after irradiation. CS treatment was found to promote an antiapoptotic gene expression profile within the intestinal epithelium, which could explain some of the radioprotection conferred by early CS treatment. Conversely, the delayed MP treatment was likely administered too late to exert a beneficial antiapoptotic response, and instead, the antiproliferative effects of MP were dominant, reducing the regenerative response and potentially magnifying the tissue injury. Therefore, the timing of CS administration may be an underappreciated, yet important, consideration for successful clinical therapeutic use of CS.

Patients with acute GVHD who demonstrate progression of disease by day 3 or no response by day 7 after CS initiation are considered to have SR GVHD (10). Patients with SR acute GVHD have a poor prognosis and limited therapeutic options (36). Given this clinical need, substantial efforts have been made to establish experimental models of SR GVHD (17, 18, 37–39). In our study, we developed 2 distinct SR GVHD models: an ex vivo coculture system using intestinal organoids cultured with GR-deficient T cells and an in vivo BMT model of moderate GVHD severity with daily MP treatment initiated after the onset of symptoms. We sought to use these SR GVHD models to discriminate the effects of CS on the intestinal epithelium from effects on the immune system, and we found that CS administration directly suppressed epithelial regeneration and, in some cases, exacerbated intestinal tissue damage. Our results are consistent with findings from patient biopsies that identified transcriptomic evidence for reduced proliferation and wound healing in intestinal tissue from patients being evaluated for SR GVHD in comparison with tissue taken at the onset of GVHD (40). Additionally, negative effects of CS on tissue recovery during immune-mediated injury are not exclusive to the GI tract, as a study examining skin GVHD found that highly potent topical CS could increase loss of hair follicles and hair follicle stem cells (41).

We found that both pharmacologic inhibition and genetic ablation of STAT3 attenuated the effects of MP on organoid growth, indicating that CS may inhibit proliferation by restraining STAT3 function. Given that IL-22–induced phosphorylation of STAT3 appeared intact in the presence of CS, this may indicate that CS interfere with STAT3’s downstream transcriptional function rather than its activation. It has been shown in human and murine cell lines that GR can bind to STAT3 and modulate STAT3-dependent transcription (33). In addition, STAT3 binding to the GR can modulate GR-dependent transcription (33, 42). Thus, there is an active interplay between these 2 molecules, and our findings indicate that this interplay may restrict STAT3-dependent epithelial proliferation but that the restriction can be overcome by augmenting activation of the JAK/STAT pathway. Further studies are needed to better understand how STAT3-GR interactions may regulate proliferation and whether IL-22 administration can overcome CS suppression by increasing the activity of STAT3 or by activating other signaling pathways.

The balance between immunologic activity and tissue tolerance may regulate the severity of tissue damage in GVHD (43). CS treatment can reduce both the immune response and the tissue’s tolerance of this response. Therefore, we attempted to increase the resilience of the tissue without forgoing the immunosuppressive benefits of CS by coadministering IL-22. This cytokine has emerged as a physiologic regulator of the intestinal epithelium after damage, promoting epithelial regeneration and protecting the intestinal mucosa from multiple types of insults, including radiation injury, genotoxic stress, infection, and T cell–mediated tissue damage (22, 34, 35, 44, 45). The use of exogenous IL-22 after transplantation is supported by the findings that host-derived IL-22–producing lymphocytes are eliminated by the donor’s immune system in GVHD (34, 46, 47) and that CS suppress IL-22 production in PBMCs (48). Further supporting this approach, in the settings of TBI and GVHD, we found that IL-22 administration could overcome the tissue-compromising effects of CS exposure and bolster intestinal crypt regeneration.

SR GVHD is a complex process with multiple potential contributory etiologies, including the responses of both the donor immune system and host tissues. This complexity is highlighted by a recent study reporting that IL-22 produced by donor T cells can worsen colon pathology in a SR GVHD model using high-dose DEX treatments (17). The distinct results in that study and ours likely relate to the numerous differences between the distinct models, including a roughly 15-fold greater effective steroid dose used in that study. Additionally, the negative effects of IL-22 identified in that study were mediated by allogeneic donor T cells, which concurrently produce multiple cytokines, potentially leading to several overactivated signaling pathways (17). In contrast, our studies focused on exogenous IL-22 administration, in which we delivered potent levels of this 1 cytokine specifically. Consistent with this approach, a recent phase II clinical trial provides evidence that combining IL-22 administration with systemic steroids may lead to improved treatment responses in patients with newly diagnosed lower-GI acute GVHD (35). Therefore, IL-22 administration may provide a complementary treatment option countering the negative tissue-specific effects of CS without inhibiting the beneficial antiinflammatory and immunosuppressive effects of CS on immune cells lacking the IL-22 receptor. Such strategies combining immunosuppression and regenerative medicine have the potential to provide a more comprehensive approach for improving intestinal recovery and treatment responses in GVHD.

## Methods

### Sex as a biological variant.

Material from both male and female mice was used for organoid experiments and for the preparation of allografts, and both males and females were included as BMT recipients (see *BMT* below for further details). Therefore, the findings are expected to be relevant to both males and females, although no experiments were performed to test for differences between the sexes.

### Mice.

C57BL/6 (B6), BALB/c, and B10.BR mice were purchased from The Jackson Laboratory (JAX). *Lgr5-LacZ* B6, *Lgr5-GFP-IRES-CreERT2* (*Lgr5-GFP*) B6, and *Olfm4-GFP-IRES-CreERT2* (*Olfm4-CreERT2*) B6 mice were provided by Hans Clevers (Hubrecht Institute, Utrecht, Netherlands). *Nr3c1^fl/fl^* *Olfm4-CreERT2* mice were created by crossing *B6.Cg-Nr3c1^tm1.1Jda^/J* mice (*Nr3c1^fl/fl^*, The Jackson Laboratory [JAX]) with *Olfm4-CreERT2* mice. *Nr3c1^fl/fl^* *Cd4-Cre* mice were created by crossing *Nr3c1^fl/fl^* mice with *B6.Cg-Tg(Cd4-Cre)1Cwi/BfluJ* mice (JAX). *Stat3^fl/fl^* *Villin-Cre* mice were created by crossing *B6.129S1-Stat3^tm1Xyfu^/J* mice (JAX) with *B6.Cg-Tg(Vil1-Cre)997Gum/J* mice (JAX). Mice were housed in a pathogen-free facility and received standard chow and autoclaved sterile drinking water. Six- to 12-week-old mice were used in the experiments.

### Crypt isolation and cell dissociation.

Isolation of intestinal crypts and the dissociation of cells were performed as previously described (22). Briefly, harvested SIs were opened longitudinally, washed in PBS, diced, and then incubated at 4°C in 10 mM EDTA with shaking for 20 minutes. Crypts were then dislodged with vigorous shaking. Crypts were incubated in 1× TrypLE Express (Gibco, Thermo Fisher Scientific) at 37°C for 10–15 minutes with mechanical disruption to obtain a single-cell suspension.

### Organoid and ISC colony culture.

For mouse organoids, 50–100 crypts were plated in 10 μL drops containing a 2:1 ratio of Matrigel (Corning) and ENR medium consisting of advanced DMEM/F12 media (Gibco, Thermo Fisher Scientific) supplemented with 2 mM GlutaMAX (Invitrogen, Thermo Fisher Scientific), 10 mM HEPES (MilliporeSigma), 100 U/mL penicillin, 100 μg/mL streptomycin (MilliporeSigma), 1× N2 supplement (Invitrogen, Thermo Fisher Scientific), 50 ng/mL mouse EGF (Peprotech), 100 ng/mL mouse noggin (Peprotech), and 5%–10% R-spondin-1–conditioned medium (CM) from R-spondin-1–transfected HEK293T cells provided by Calvin J. Kuo (Stanford University, Stanford, California, USA) (49). Once drops polymerized, cultures were maintained in ENR medium that was replaced every 2–3 days.

For experiments with *Nr3c1^–/–^* organoids, *Nr3c1^ΔIEC^* mice, and littermate controls received daily i.p. injections of tamoxifen (2 mg, MilliporeSigma, suspended in sunflower oil) for 5 consecutive days prior to crypt isolation and organoid culturing.

ISC colonies were generated with ISCs sorted from *Lgr5-GFP* mice (24). Next, 3,000 ISCs were plated in 10 μL Matrigel drops and cultured in ENR medium with CHIR99021 (3 μM, Stemgent) and valproic acid (1.5 mM, MilliporeSigma). The medium also contained the Rho-kinase/ROCK inhibitor Y-27632 (10 μM, Tocris) and jagged 1 (1 μM, Anaspec) for the first 48 hours of culturing.

Cell cultures were treated with MP (Tocris), DEX (Tocris), or budesonide (Tocris) as well as rmIFN-γ (R&D Systems), rmIL-22 (R&D Systems), ruxolitinib (Selleckchem), or Stattic (Tocris).

As described previously (30), for coculturing of intestinal organoids with T cells, CD5^+^ T cells were isolated from splenocytes using magnetic MicroBeads with the MACS system (Miltenyi Biotec). T cell activation was achieved by culturing cells with 5 μg/mL plate-bound anti-CD3 mAb (BD Biosciences, 145-2C11) and 2 μg/mL anti-CD28 mAb (BD Biosciences, 37.51). After 3–5 days of culturing, activated T cells and freshly isolated SI crypts were cultured in Matrigel at a 500:1 T cell/crypt ratio.

Healthy human duodenal organoids were cultured from banked frozen organoids (> passage 7) that had been previously generated from biopsies obtained during duodenoscopy of healthy human controls. Organoids were passaged via single-cell dissociation using 1× TrypLE Express (Gibco, Thermo Fisher Scientific) and resuspended in medium without growth factors (GF^–^) that was composed of advanced DMEM/F12 (Gibco, Thermo Fisher Scientific), 100 U/mL penicillin-streptomycin (Gibco, Thermo Fisher Scientific), 10 mM HEPES (Gibco, Thermo Fisher Scientific), GlutaMAX (Gibco, Thermo Fisher Scientific), and 50%–66% Matrigel (Corning). After plating and Matrigel polymerization, human SI organoid expansion medium was added and consisted of GF^–^, Wnt CM (50% final concentration), R-spondin CM (20% final concentration), noggin CM (10% final concentration), 50 ng/mL murine EGF (Peprotech), 10 mM nicotinamide (MilliporeSigma), 1.25 mM *N*-acetylcysteine (MilliporeSigma), 1× B27 (Gibco, Thermo Fisher Scientific), 500 nM TGF-β inhibitor A83-01 (Tocris), 10 μM P38 inhibitor SB202190 (MilliporeSigma), and 100 μg/mL Primocin (optional) (Invitrogen, Thermo Fisher Scientific). The medium was refreshed every 2–3 days. Along with medium changes, treatment wells received different concentrations of MP (Pfizer) or rhIFN-γ (R&D Systems). RhIL-22 (Genscript) was added daily.

### Organoid irradiation.

Irradiation was delivered with a Shepherd Mark-I Unit (model 68, SN643, J.L. Shepherd & Associates) operating a ^137^Cs source. Following isolation, SI crypts were plated at a density of 50–100 crypts/well and incubated for 4 hours before being irradiated (4 Gy).

### Imaging of organoids and colonies.

Random representative nonoverlapping images of organoids and colonies were acquired from each well using an Axio Observer Z1 inverted microscope (Carl Zeiss). Additional images were acquired with a Cytation 7 Cell Imaging Multimode Reader (Biotek) using z-stack tile imaging. Human SI organoid images were acquired with an EVOS FL Cell Imaging System (Thermo Fisher Scientific). Organoid counts and size were analyzed using ImageJ (NIH).

### TBI.

Mice were exposed to 10 Gy radiation using a GammaCell 40 irradiator (Best Theratronics Ltd.).

### BMT.

MHC-mismatched BMTs were performed in 8- to 10-week-old mice as previously described (50). In the B6-into-BALB/c (H-2^b^ into H-2^d^) transplantations, female mice were used as donors and recipients, and the recipient mice were conditioned with an 850 cGy split dose of radiation prior to BMT. In B10.BR-into-B6 BMT (H-2^k^ into H-2^b^) transplantations, male mice were used as donors, males and females were used as recipients, and the recipient mice were irradiated with a split dose of 1,100 cGy. BM cells were obtained from femurs and tibias aseptically by washing BM canals with medium. BM cells were T cell depleted (TCD) using an anti-Thy 1.2 Ab (Bio X Cell, 30H12) and low-TOX-M rabbit complement (Cedarlane Laboratories). Splenic donor T cells were isolated as described earlier. For experiments depicted in Figures 5, A–G, and Figure 6, N–Q, as well as in Supplemental Figure 1, BALB/c recipients received 5 × 10^6^ TCD BM cells with or without 0.5 × 10^6^ T cells per mouse via tail vein injection. B6 recipients received 5 × 10^6^ TCD BM cells and 1 × 10^6^ T cells from B10.BR or B6 donors via tail vein injection. Mice were monitored daily for survival and weekly with an established clinical GVHD scoring system examining weight, posture, activity, fur ruffling, and skin integrity (51). *Nr3c1^ΔIEC^* mice (and littermate controls) were given daily i.p. injections of tamoxifen (2 mg/mouse, dissolved in sunflower oil, MilliporeSigma) for 5 consecutive days prior to BMT.

For the survival experiments depicted in Figure 1A, BALB/c mice were irradiated at 700 cGy on day –1, followed by i.v. transplantation with 1 × 10^7^ non-TCD BM cells with or without 2 × 10^6^ purified T cells from B6 donors on day 0. Donor T cells were purified from splenocytes using biotin-labeled Abs against CD19 (1D3), CD45R (RA3–6B2), CD11b (M1/70), CD11c (N418), CD49b (DX5), NK1.1 (PK136), TCR γΔ (GL3), and TER-119 (TER-119) from STEMCELL Technologies, followed by streptavidin RapidSpheres depletion with an EasySep magnet, also from STEMCELL Technologies. Survival was monitored daily, and weights and clinical scores were recorded twice per week.

### In vivo treatment.

Prednisolone (Merck) was administered by i.p. injection at a daily dose of 1, 3, or 6 mg/kg, starting on day 1 and continuing until day 28 after BMT. MP (Tocris) was administered by i.p. injection at a daily dose of 2 mg/kg at the indicated time points. F-652 (Evive Biotech, formerly Generon) was administered s.c. at a daily dose of 100 μg/kg at the indicated time points. The control groups received vehicle alone.

### GVHD histopathology.

Mice were euthanized for histopathologic analysis at the indicated time points after BMT. The SIs were formalin preserved, paraffin embedded, sectioned, and stained with H&E (Vector Laboratories). GVHD histopathology assessment was performed by a pathologist blinded to the treatment groups. As described previously (51), tissues were analyzed using a semiquantitative score based on 19 parameters associated with GVHD, with a maximum possible score of 3 for each parameter.

### LacZ staining.

SIs were collected from *Lgr5-LacZ* mice. β-Galactosidase (LacZ) staining was performed as previously described (2). Washed 2.5 cm SI fragments were incubated with an ice-cold fixative consisting of 1% formaldehyde, 0.02% igepal, and 0.2% glutaraldehyde. After removing the fixative, organs were stained for the presence of LacZ according to the manufacturer’s protocol (LacZ Staining Kit, InvivoGen). The organs were then formalin preserved, paraffin embedded, sectioned, and counterstained with Nuclear Fast Red (Vector Laboratories). The distance between the Lgr5^+^ crypt region and the beginning of the villus was measured to evaluate the size of the TA compartment.

### IHC staining.

Formalin-fixed tissue sections were deparaffinized with SafeClear II (Thermo Fisher Scientific), followed by rehydration in an ethanol gradient. Sections were incubated in antigen retrieval buffer (10 mM sodium citrate, pH 6, 0.05% Tween-20) in a steamer for 40 minutes, followed by blocking with 1.5% goat serum for 60 minutes at room temperature. Slides were incubated with anti-Ki67 Ab (Abcam, Ab15580, 1:800) or anti-Olfm4 Ab (Cell Signaling Technology, 39141, 1:200) in 2.5% goat serum at 4°C overnight. Tissue was then stained with the anti–rabbit IgG ABC Kit (Vector Laboratories), and immunostaining was detected using an ImmPACT AMEC Red Peroxidase (HRP) Substrate Kit (Vector Laboratories) according to the supplier’s instructions. Sections were counterstained with hematoxylin QS (Vector Laboratories) and coverslipped with VectaMount (Vector Laboratories).

### Flow cytometry.

Mouse organoids were dissociated using 1× TrypLE Express (Gibco, Thermo Fisher Scientific) at 37°C with mechanical disruption. Cells were then washed in DMEM/F12 medium with 10% FBS and 2 kU/mL DNase1 and then passed through a 40 μm cell strainer. Cells were incubated with the Fixation/Permeabilization kit (eBioscience), followed by anti-Ki67 (BioLegend, Ki-67), anti-GFP (Invitrogen, Thermo Fisher Scientific, A-21311), and DAPI staining.

For the CTV cell proliferation assay, human organoids were dissociated into single cells and stained with CTV (Invitrogen, Thermo Fisher Scientific, 5 μM in PBSO) before plating. After 5 days of culturing, organoids were harvested, processed into single cells, stained with the live/dead marker Zombie NIR (BioLegend), and analyzed by flow cytometry for CTV MFI in live cells.

For flow cytometry of splenocytes, spleens were mashed into single-cell suspensions and treated with ACK lysing buffer (room temperature, 5 min, Thermo Fisher Scientific). After thorough washing, cells were stained with Abs targeting CD4 (BioLegend, RM4-5), CD8 (BioLegend, 53–6.7), CD45 (BioLegend, 30-F11), CD44 (eBioscience IM7), and CD62L (Invitrogen, Thermo Fisher Scientific, MEL-14). Fixable Live/Dead Cell Stain Kits (Invitrogen, Thermo Fisher Scientific) were also used. Flow cytometry was performed with BD (LSR II and LSRFortessa X-50 with FACSDiva software) and Beckman Coulter (CytoFLEX with CytExpert software) cytometers, and the data were analyzed with FlowJo software (TreeStar).

### RT–qPCR.

RNA was isolated from organoids or SI crypts with TRIZol reagent (Invitrogen, Thermo Fisher Scientific) and an E.Z.N.A. Total RNA Kit I (Omega-Biotek). Reverse transcription of RNA was performed using the High-Capacity cDNA Reverse Transcription Kit (Applied Biosystems). Specific primers were obtained from Applied Biosystems: *Gapdh*: Mm99999915_g1; *Cdkn1a*: Mm00432448_m1. Other primer sequences were obtained from PrimerBank: *Gapdh* (ID 6679937a1), *Cdkn1a* (ID 6671726a1), *Ccna2* (ID 6753308a1), *Ccnb1* (ID 28195398a1), *Bcl2l1* (ID 31981887a1), *Bcl2* (ID 28916685a1), *Bak1* (ID 15553445a1), and *Bik* (ID 6671638a1). cDNA and primers were mixed with either TaqMan or PowerTrack SYBR Master Mix (Applied Biosystems) and reverse transcription quantitative PCR (RT-qPCR) was performed on a QuantStudio 7 Flex System (Applied Biosystems). Relative expression was calculated by the comparative ΔCt method with *Gapdh*.

### Western blotting.

Organoids were collected in RIPA buffer (Thermo Fisher Scientific) containing protease and phosphatase inhibitors (Thermo Fisher Scientific) and incubated for 15 minutes on ice. Samples were sonicated and centrifuged at 14,000*g*, and protein-containing supernatant was collected.

For Western blotting, protein samples were boiled at 90°C for 10 minutes with sample buffer and 0.1 M DTT (Thermo Fisher Scientific). Samples were then run through SDS-PAGE, followed by transfer to nitrocellulose membranes. Protein-bound membranes were incubated in a blocking buffer (2% milk and 0.2% Tween-20 in PBS, or 2% BSA in PBS) for 1 hour at room temperature. Membranes were then incubated overnight at 4°C with rabbit IgG mAbs (Cell Signaling Technology) targeting GAPDH (5174, 1:2,000), STAT3 (12640, 1:2,000), and phosphorylated STAT3 (p-STAT3) (9145, 1:1,000). Membranes were then washed with PBST (PBS with 0.2% Tween-20) followed by incubation with an anti–rabbit IgG HRP-linked secondary Ab (Cell Signaling Technology, 7074, 1:1,000) for 1 hour at room temperature. After another wash in PBST, membranes were incubated for 2 minutes with Pierce ECL Western Blotting Substrate (Thermo Fisher Scientific). Chemiluminescence was detected using autoradiography film (Thermo Fisher Scientific).

### Computational analyses.

SI epithelial cells from WT B6 mice (GEO GSE92332) (19) were analyzed using Scanpy (52, 53). The original data set consists of 6 batch samples encompassing 13,353 cells and 27,998 genes. Cells with fewer than 700 genes, fewer than 1,500 or more than 40,000 unique molecular identifier (UMI) counts per cell, and a mitochondrial gene expression fraction higher than 0.2 were removed. Genes detected in fewer than 20 cells were excluded. After filtering, a total of 12,457 cells and 12,818 genes remained. The filtered data set was normalized to the median library size and log transformed. ComBat batch correction was then applied (54), after which 4,000 highly variable genes were identified and extracted. The normalized expression levels then underwent linear regression to remove the effects of total reads per cell and cell-cycle genes, followed by a *z* transformation. Dimension reduction was performed using principal component analysis (PCA) and then uniform manifold approximation and projection (UMAP) on the top 50 principal components (PCs) and 30 nearest neighbors (55). For de-noising and imputation, we used the MAGIC algorithm (56). Clustering was calculated using the Louvain algorithm within Scanpy with 0.5 resolution (57). Detected clusters were mapped to cell types or intermediate states using markers for intestinal epithelial cell subtypes (19).

Analysis of a published scRNA-Seq data set (GEO GSE119969, GSM3389578 Human_SI_tissue) of human ileum epithelial crypt cells was based on the provided methodology (23) using the R package Seurat (58–61). In brief, cells with fewer than 2.5% or more than 15% UMI reads mapped to mitochondrial genes and fewer than 200 expressed genes were removed. Genes expressed in fewer than 3 cells were excluded. A total of 2,342 cells and 17,562 genes were processed for downstream analysis. The expression matrix was log normalized by the NormalizeData function. Using the ScaleData function, total UMI counts per cell and proportions of mitochondrial reads were corrected by negative binomial regression. Variably expressed genes were identified with the FindVariableFeatures function, with scaled dispersion above 0.5 and log-normalized average expression between 0.125 and 3, and were used to perform linear dimensionality reduction with RunPCA. Subsequently, cell clusters were identified with the FindNeighbors and FindClusters functions using the top 25 PCs with the following parameters: k.param = 20, prune.SNN = 1/15 and resolution 0.6. For visualization in 2 dimensions, UMAP plots were generated with the RunUMAP function using the top 25 PCs. With the FindAllMarkers function, differentially expressed genes (Wilcoxon rank-sum test) in each cluster were identified with at least a 0.25 log-fold increase. Markers for intestinal epithelial cell types were used to assign cell identity to the clusters (23). The cluster of immune cells, also described in the original manuscript, was excluded using the Subset function in the Seurat package, after which all previous steps of analysis were repeated from the identification of variably expressed genes until cluster identification, using the same parameters on the remaining 2,262 cells. Finally, the MAGIC function in the Rmagic package was used for de-noising and imputation, after which genes were projected on the UMAP plots (56).

For analysis of a published RNA-Seq data set of mouse intestinal epithelium after DEX treatment (GEO GSE113691), GSEA software, version 4.2.1 (62, 63) was used. After quantile normalization, GSEA was performed between DEX-treated and control samples using KEGG and MSigDB Hallmarks pathways. Gene sets of interest with a nominal *P* value of less than 0.05 and an FDR of less than 0.25 were visualized as an enrichment plot.

### Statistics.

To detect an effect size of greater than 50% difference in means, with an assumed coefficient of variation of 30%, which is common in biological systems, we attempted to have at least 5 samples per group, particularly for in vivo studies. Systemic measures from in vivo studies were reported for individual mice. Organoid quantifications were based on the average measurement of individual organoids in each culture well. Histological analysis of crypt numbers, crypt height, and the crypt/villus height ratio was performed on and averaged for independent ileal cross sections, with data pooled from multiple mice. Quantification of cell numbers (ISCs, Ki67^+^ cells, and Olfm4^+^ cells) in individual crypts was averaged and reported for each cross section evaluated. No data were excluded from the study. All experiments were repeated at least once, unless otherwise stated.

All statistical tests were 2 sided. A 2-tailed *t* test or 1-way ANOVA was performed for comparison of 2 or multiple groups, respectively. Adjustments for multiple comparisons were made. All analyses of statistical significance were calculated and are displayed compared with the reference control group unless otherwise stated. A *P* value of less than 0.05 was considered statistically significant. Graphs show the mean ± SEM for each group unless stated otherwise. Statistical analyses and graphs were generated using GraphPad Prism, version 10.1.1 (GraphPad Software).

### Study approval.

All animal experiments were performed in accordance with the institutional protocol guidelines of the IACUC of Memorial Sloan Kettering Cancer Center (MSKCC). Human organoid studies were performed with samples from patients who provided informed consent and were approved by the IRB of UMC Utrecht (Utrecht, the Netherlands; METC 10-402/K; TCBio 19-489).

### Data availability.

All supporting data from the study can be found in the Supporting Data Values file. Code from computational analyses can be obtained upon request from the corresponding author.

## Author contributions

VA and WYC designed, performed, and analyzed in vivo and ex vivo experiments and drafted the manuscript. SAJ designed, performed, and analyzed human ex vivo experiments. GT designed, performed, and analyzed survival experiments. MC and ST provided input, designed and performed experiments, and helped with various assays. PV, YF, and TI performed and analyzed in vivo experiments. AE and JK performed BMTs, monitored BM transplants, and maintained the mouse colonies. AP performed and analyzed ex vivo experiments. MVH performed and analyzed human ex vivo experiments. CL analyzed intestinal histopathology. BRB, CAL, and AMH supervised the research.

## Supplementary Material

Supplemental data

Unedited blot and gel images

Supporting data values

## Version 1 02/13/2024

In-Press Preview

## Version 2 04/01/2024

Electronic publication
