# Supplementary material for: Corticosteroids impair epithelial regeneration in immune-mediated intestinal damage
Source: J Clin Invest. 2024 Feb 13;134(7):e155880. doi: 10.1172/JCI155880 (PMC10977993; doi:10.1172/JCI155880)
Supplement: Supplemental data [file jci-134-155880-s105.pdf]

Supplementary Figures for

Corticosteroids impair epithelial regeneration in immune-mediated intestinal damage.

Viktor Arnhold, Winston Y. Chang, Suze A. Jansen, Govindarajan Thangavelu, Marco Calafiore, Paola Vinci, Ya-Yuan Fu, Takahiro Ito, Shuichiro Takashima, Anastasiya Egorova, Jason Kuttiyara, Adam Perlstein, Marliek van Hoesel, Chen Liu, Bruce R. Blazar, Caroline A. Lindemans, Alan M. Hanash

Correspondence to:

Alan M. Hanash
hanasha@mskcc.org
1275 York Ave.
New York, NY 10065
P: 646-888-3437
F: 646-422-0298

Supplemental Figure 1

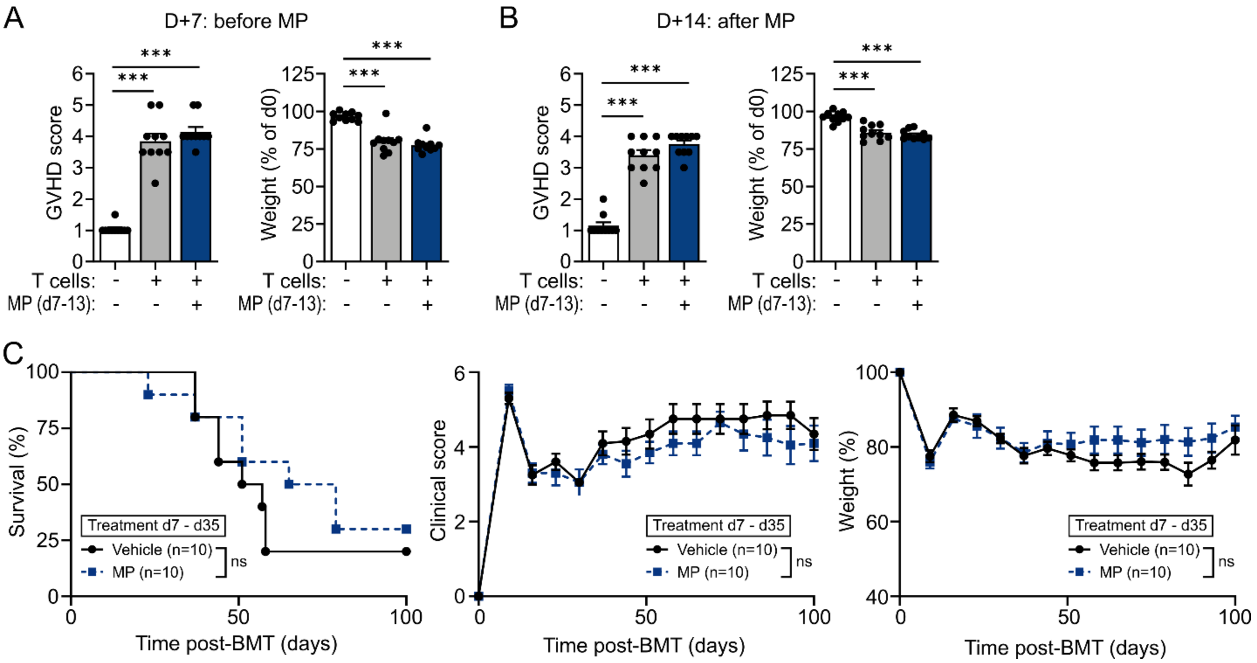

Supplemental Figure 1. Systemic signs of GVHD after allo-BMT with or without corticosteroid treatment starting day 7 post-BMT. (**A**-**B**) B6-into-BALB/c transplant of BM with or without T cells. Recipients were treated with methylprednisolone (MP; 2 mg/kg) or vehicle i.p. daily starting on day 7 after BMT. GVHD score and body weight, 7 and 14 days after BMT (n = 10 mice per group). (**C**) Percentage survival, clinical score of GVHD, and relative weight of recipients in a B6-into-BALB/c transplant of BM and T cells with or without MP treatment (2 mg/kg, i.p. daily) from day 7 to 35 post-BMT (n = 10 mice per group). Statistics performed with 1-way ANOVA (A and B) or log–rank analysis (C); NS, not significant; ***P < 0.001. Data are combined from 2 experiments (A and B) or representative of at least 2 independent experiments (C).

Supplemental Figure 2

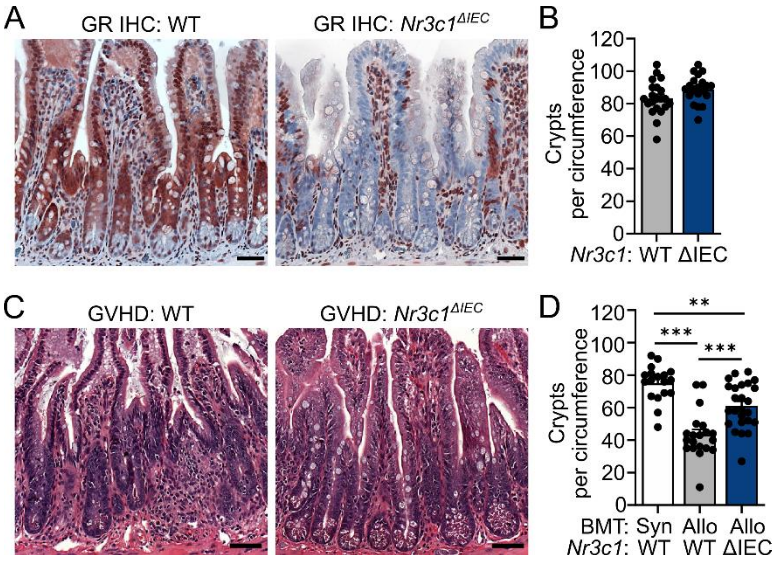

Supplemental Figure 2. Intestinal epithelium-specific GR deletion reduces GVHD-associated intestinal injury. (**A**-**B**) Representative glucocorticoid receptor (GR) IHC images and ileal crypt frequency of *Nr3c1*$^{fl/fl}$ *Olfm4-CreERT2* (*Nr3c1*$^{\Delta IEC}$) mice and WT littermates after tamoxifen treatment (n = 20-21 sections per group). Scale bars: 50 µm. (**C**-**D**) B10.BR-into-B6 transplant of syngeneic or allogeneic BM and T cells with WT and *Nr3c1*$^{\Delta IEC}$ recipients. Representative images and ileal crypt frequency on day 14 post-BMT (n = 18-25 sections per group). Scale bars: 50 µm. Statistics performed with 2-sided t test or 1-way ANOVA; **P<0.01, ***P < 0.001. Data are combined from 2 experiments.

Supplemental Figure 3

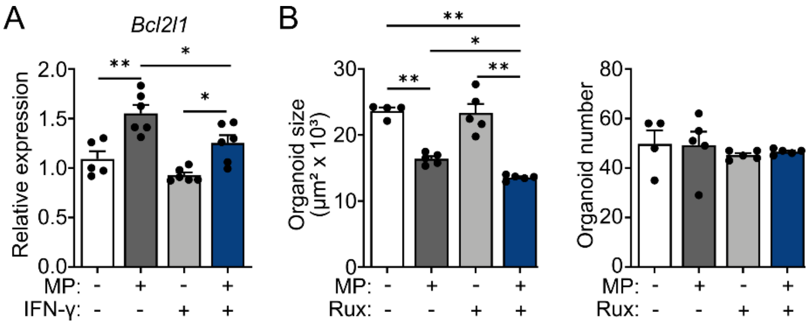

Supplemental Figure 3. Ruxolitinib treatment does not prevent corticosteroid-mediated suppression of organoid growth. (**A**) RT-qPCR of *Bcl2l1* in SI organoids cultured with or without MP (10 μM) and rmIFN-γ (1 ng/ml) for 3 days (n = 5-6 wells per group). (**B**) Size of SI organoids cultured with or without MP (10 μM) and ruxolitinib (10 nM) for 3 days (n = 4-5 wells per group). Statistics performed with 1-way ANOVA; *$P < 0.05$, **$P < 0.01$. Data are representative of at least 2 independent experiments.

Supplemental Figure 4

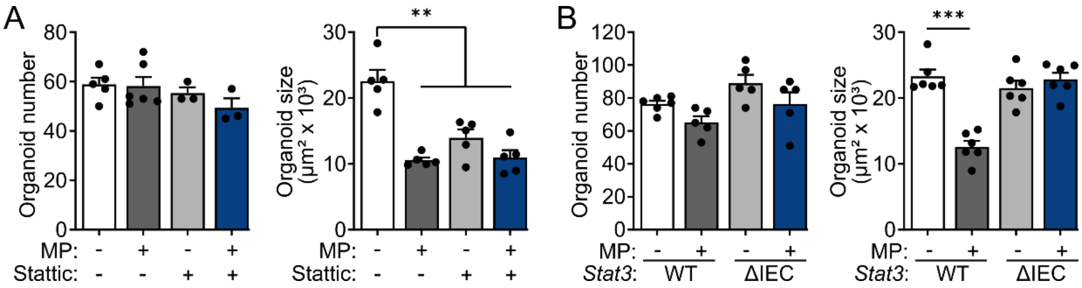

Supplemental Figure 4. STAT3 inhibition during corticosteroid-mediated suppression of ex vivo organoid growth*.* (**A**) Frequency and size of SI organoids cultured with or without MP (10 μM) and Stattic (50 μM) for 5 days (*n* = 3-6 wells per group). (**B**) Frequency and size of *Stat3*$^{fl/fl}$ *Villin-Cre* (*Stat3*$^{\Delta IEC}$) and WT SI organoids cultured with or without MP (10 μM) for 5 days (*n* = 5-6 wells per group). Statistics performed with 1-way ANOVA; $^{**}P < 0.01$, $^{***}P < 0.001$. Data are representative of 2 independent experiments.

Supplemental Figure 5

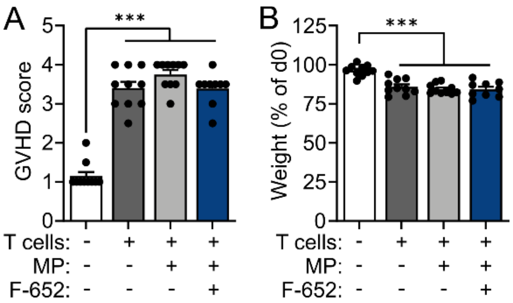

Supplemental Figure 5. Systemic signs of GVHD after treatment with corticosteroids and F-652 starting day 7 post-BMT. (**A**-**B**) B6-into-BALB/c transplant of BM with or without T cells. Recipients were treated with or without MP (2 mg/kg i.p. daily) and with or without F-652 (100 μg/kg s.c. every other day). Shown are the clinical GVHD score and body weight loss 14 days post-BMT (*n* = 9-10 mice per group). Statistics performed with 1-way ANOVA; ***P < 0.001. Data are combined from 2 independent experiments.
