# Supplementary material for: Corticosteroids impair epithelial regeneration in immune-mediated intestinal damage
Source: J Clin Invest. 2024 Feb 13;134(7):e155880. doi: 10.1172/JCI155880 (PMC10977993; doi:10.1172/JCI155880)

Full unedited gel for Figure 6A

Proteins detected:
p-STAT3
STAT3
GAPDH

**Bands used in figure

Lane #:
1: Untreated
2: rmIL-22
3: MP
4L rmIL-22 & MP

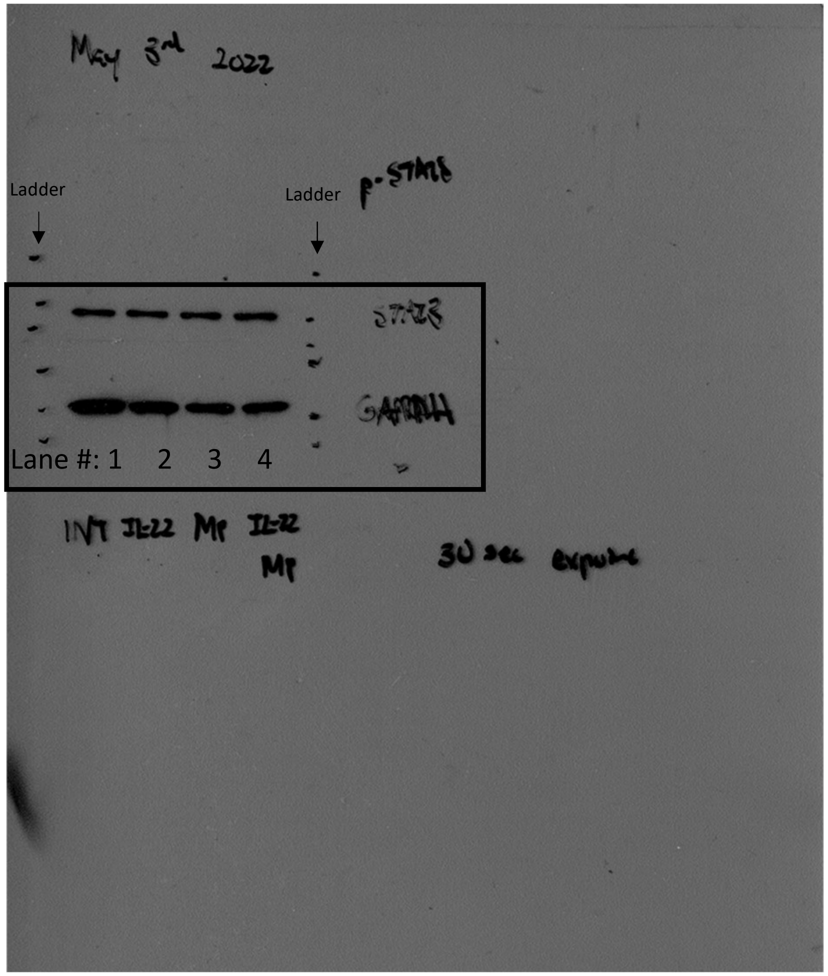

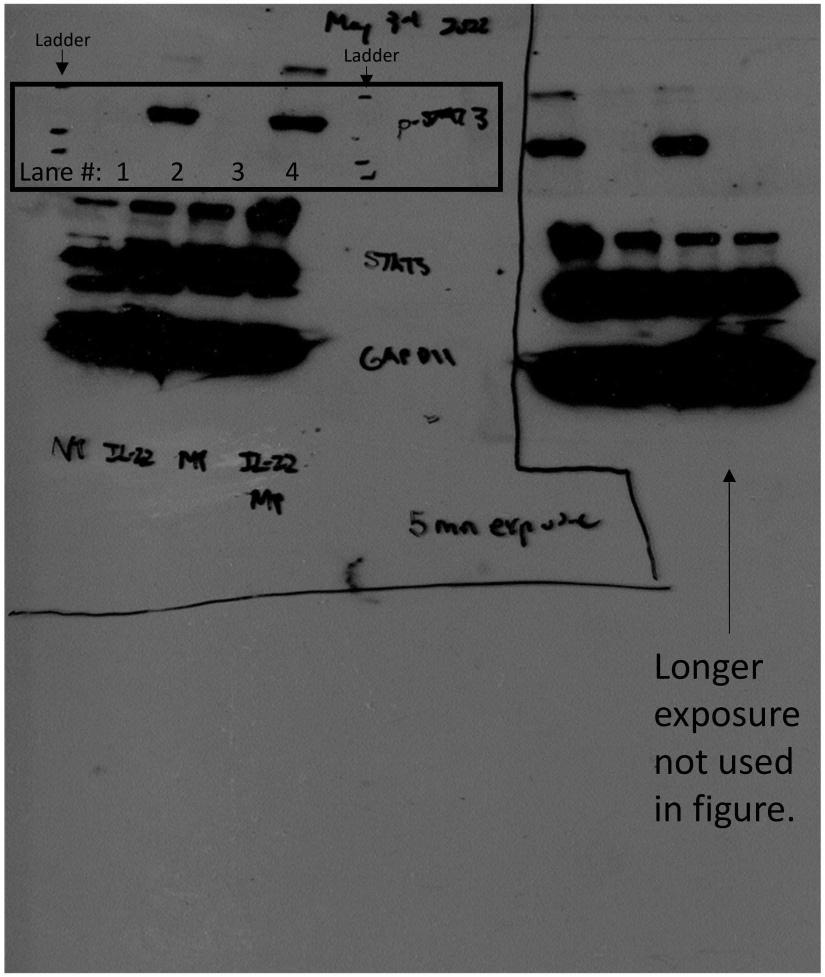

Supplement: Unedited blot and gel images [file jci-134-155880-s106.pdf]
